# Assessment of Clinical Trials Supporting US Food and Drug Administration Approval of Novel Therapeutic Agents, 1995-2017

**DOI:** 10.1001/jamanetworkopen.2020.3284

**Published:** 2020-04-21

**Authors:** Audrey D. Zhang, Jeremy Puthumana, Nicholas S. Downing, Nilay D. Shah, Harlan M. Krumholz, Joseph S. Ross

**Affiliations:** 1 New York University School of Medicine, New York; 2 Center for Outcomes Research and Evaluation, Yale–New Haven Hospital, New Haven, Connecticut; 3 Yale School of Medicine, New Haven, Connecticut; 4 Brigham and Women’s Hospital, Boston, Massachusetts; 5 now with Bain Capital Life Sciences, Boston Massachusetts; 6 Division of Health Care Policy and Research and Robert D. and Patricia E. Kern Center for the Science of Health Care Delivery, Mayo Clinic, Rochester, Minnesota; 7 Section of Cardiovascular Medicine, Yale School of Medicine, New Haven, Connecticut; 8 Department of Health Policy and Management, Yale School of Public Health, New Haven, Connecticut; 9 Section of General Internal Medicine, Yale School of Medicine, New Haven, Connecticut; 10 National Clinician Scholars Program, Yale School of Medicine, Department of Internal Medicine, New Haven, Connecticut

## Abstract

**Importance:**

Since the introduction of the Fast Track designation in 1988, the number of special regulatory programs available for the approval of new drugs and biologics by the US Food and Drug Administration (FDA) has increased, offering the agency flexibility with respect to evidentiary requirements.

**Objective:**

To characterize pivotal efficacy trials supporting the approval of new drugs and biologics during the past 3 decades.

**Design, Setting, and Participants:**

This cross-sectional study included 273 new drugs and biologics approved by the FDA for 339 indications from 1995 to 1997, from 2005 to 2007, and from 2015 to 2017.

**Main Outcomes and Measures:**

Therapeutics were classified by product type and therapeutic area as well as orphan designation and use of special regulatory programs, such as Priority Review and Accelerated Approval. Pivotal trials were characterized by use of randomization, blinding, types of comparators, primary end points, number of treated patients, and trial duration, both individually and aggregated by each indication approval.

**Results:**

A total of 273 new drugs and biologics were approved by the FDA in these 3 periods (107 [39.2%] in 1995-1997; 57 [20.9%] in 2005-2007; and 109 [39.9%] in 2015-2017), representing 339 indications (157 [46.3%], 64 [18.9%], and 118 [34.8%], respectively). The proportion of therapeutic approvals using at least 1 special regulatory program increased (37 [34.6%] in 1995-1997; 33 [57.9%] in 2005-2007; and 70 [64.2%] in 2015-2017), as did indication approvals receiving an orphan designation (20 [12.7%] in 1995-1997; 17 [26.6%] in 2005-2007, and 45 [38.1%] in 2015-2017). The most common therapeutic areas differed over time (infectious disease, 53 [33.8%] in 1995-1997 vs cancer, 32 [27.1%] in 2015-2017). When considering the aggregate pivotal trials supporting each indication approval, the proportion of indications supported by at least 2 pivotal trials decreased (80.6% [95% CI, 72.6%-87.2%] in 1995-1997; 60.3% [95% CI, 47.2%-72.4%] in 2005-2007; and 52.8% [95% CI, 42.9%-62.6%] in 2015-2017; *P* < .001). The proportion of indications supported by only single-group pivotal trials increased (4.0% [95% CI, 1.3%-9.2%] in 1995-1997; 12.7% [95% CI, 5.6%-23.5%] in 2005-2007; and 17.0% [95% CI, 10.4%-25.5%] in 2015-2017; *P* = .001), whereas the proportion supported by at least 1 pivotal trial of 6 months’ duration increased (25.8% [95% CI, 18.4%-34.4%] in 1995-1997; 34.9% [95% CI, 23.3%-48.0%] in 2005-2007; and 46.2% [95% CI, 36.5%-56.2%] in 2015-2017; *P* = .001).

**Conclusions and Relevance:**

In this study, more recent FDA approvals of new drugs and biologics were based on fewer pivotal trials, which, when aggregated by indication, had less rigorous designs but longer trial durations, suggesting an ongoing need for continued evaluation of therapeutic safety and efficacy after approval.

## Key Points

**Question:**

Have the number and characteristics of pivotal efficacy trials supporting US Food and Drug Administration approval of new drugs and biologics changed during the past 3 decades?

**Findings:**

In this cross-sectional study of 273 new drugs and biologics approved by the Food and Drug Administration for 339 indications in 3 periods (1995-1997, 2005-2007, and 2015-2017), more recent approvals increasingly used special regulatory programs and were based on fewer pivotal trials. When aggregated by indication, these trials had less rigorous designs but longer trial durations over time.

**Meaning:**

This study found changes in the evidence supporting Food and Drug Administration approval of new drugs and biologics that suggest an ongoing need for continued evaluation of therapeutic safety and efficacy after approval.

## Teaser

This cross-sectional study characterizes pivotal efficacy trials supporting US Food and Drug Administration approval of new drugs and biologics during the past 3 decades.

## Introduction

In the United States, the US Food and Drug Administration (FDA) issues approvals for new drugs and biologics that have demonstrated safety and efficacy in “adequate and well-controlled studies.”^1^ Pivotal trials are the most critical of these trials, often identified directly by FDA reviewers as the basis for approval and described in detail in FDA approval packages.^1^ Early guidance suggested that at least 2 such trials were required for approval,^2^ but the FDA has maintained a flexible interpretation, taking into consideration the ethical acceptability of conducting additional trials or the rarity of diseases when determining the sufficient threshold for safety and efficacy.^3^ As a result, the quantity and quality of evidence supporting recent drug approvals is variable, both in terms of the number of pivotal trials and their design features, such as randomization, blinding, choice of comparators and end points, number of treated patients, and trial duration.^4,5,6^

Potentially contributing to this variability is the increasing number of special regulatory programs available to the FDA during the past 30 years, now including Fast Track (1988, in statute 1997), Priority Review (1992), Accelerated Approval (1992), and Breakthrough Therapy designation (2012). Many of these programs codify special evidentiary standards acceptable for FDA approval of certain drugs and biologics, such as the use of surrogate end points (Accelerated Approval) and the acceptability of single trials as the basis of approval (Fast Track),^7,8^ with the goal of promoting earlier market availability of certain therapies, such as those addressing an unmet need or those treating serious or life-threatening conditions (eTable 1 in the Supplement). As these new programs are conformed to the regulatory environment in addition to existing programs, such as orphan designation (1983) for rare diseases,^7^ it is critical to understand their potential influence on the quality of evidence supporting the new drugs and biologics that clinicians prescribe to their patients.

To address this question, we examined the clinical evidence supporting FDA approval of new drugs and biologics in the following 3-year periods, selected to illustrate the step-wise statutory implementation of the special regulatory programs across 3 decades: 1995 to 1997, 2005 to 2007, and 2015 to 2017. We characterized all new drug and biologic approvals within each given year as well as the pivotal trials supporting these approvals and determined whether trial design features differed by time period, including use of randomization, blinding, types of comparators, types of primary end points, number of treated patients, and trial duration. These findings could offer important insights into the influence of special regulatory programs on the FDA’s evidentiary standards for new drugs and biologics over time as well as helping patients and clinicians better understand whether the clinical evidence supporting FDA approvals has changed.

## Methods

This study was prepared in accordance with the Strengthening the Reporting of Observational Studies in Epidemiology (STROBE) reporting guideline for cross-sectional studies. The study did not require institutional review board approval or patient informed consent because it was based on publicly available information and involved no patient records.

### Sample Construction

The FDA lists all new drug applications and biologic licensing applications on the Drugs@FDA database.^9^ Using a previously described method,^5^ we identified new drugs and biologics (eg, new molecular entities or new biologic drugs) approved during the periods of 1995 to 1997, 2005 to 2007, and 2015 to 2017, excluding new formulations, generics, and nontherapeutic agents (eg, diagnostic and contrast agents). We obtained the complete action package for the original approval of each therapeutic, either through the Drugs@FDA database or, for therapeutics approved from 1995 to 1997, through a Freedom of Information Act request.

### Therapeutic Indication and Regulatory Characteristics

Based on information from the approval package, we classified each novel therapeutic by period of approval and product type (small-molecule drug or biologic). Using information available in the approval packages and from public listings available on the FDA website, we identified whether each therapeutic was evaluated through a special regulatory program (Priority Review, Accelerated Approval, Fast Track, Breakthrough Therapy).^10,11,12,13^ These special regulatory programs are used for therapeutics that are intended to address unmet medical needs for serious or life-threatening conditions.^14^ Data on certain special regulatory programs were not available in all years; data for Fast Track were only available after 1997, when it was codified by the 1997 Food and Drug Administration Modernization Act, and Breakthrough Therapy was introduced in 2012 by the Food and Drug Administration Safety and Innovation Act.^15^

Using information available in the approval packages, we identified the indications for each novel therapeutic at the time of initial approval. We classified these into 1 of 8 therapeutic areas, based on the World Health Organization Anatomical Therapeutic Classification System.^16^ Using the Orphan Products Designation Database, we also determined whether these originally approved indications had been granted orphan status, a designation granted at sponsor request to drugs for indications for which there are 200 000 or fewer patients in the United States or those for which alternative therapeutic options are often not available.^17^

### Trial Characteristics

We followed a previously described method to identify and characterize the pivotal efficacy trials used as the basis of approval for each indication of each new drug or biologic (eAppendix in the Supplement).^4,5^ Briefly, these were trials explicitly labeled in FDA medical reviews as pivotal. In cases in which trials were not explicitly labeled, we first collected all trials submitted for evaluation in the FDA medical review and then identified as pivotal those trials described by FDA medical reviewers as essential to approval, those prioritized within the review with substantial discussion of study design (eg, thorough description of study protocol and inclusion and exclusion criteria), or those prioritized within the review with independent analysis of study results (ie, not pooled with other studies). Additionally, any new efficacy trial reviewed as part of a resubmitted application was considered pivotal to approval.

For each identified trial, we determined its use of randomization and blinding, categorized as randomized vs nonrandomized and double-blinded vs not double-blinded, respectively. Next, we categorized use of a comparator as active treatment, placebo control, or none. We categorized primary trial end points as clinical end points, clinical scales, or surrogate end points using a previously developed framework.^5,18^ Briefly, clinical end points, such as death or hospitalization, are those that measure patient-reported outcomes, function, or survival; clinical scales, such as the visual analog scale for pain, represent ordinal characterizations of symptoms; and surrogate end points, such as hemoglobin A_1c_ level, represent biomarkers expected to predict clinical benefit. We determined the number of treated patients by abstracting the number of patients included in intention-to-treat analyses. We also determined the duration of each trial. For time-driven end points, duration was defined as the time of primary end point measurement, such as hemoglobin A_1c_ level at 24 weeks. For event-driven end points, such as progression-free survival, duration was defined as the median follow-up time for participants or a weighted average of the median follow-up time in cases in which it differed between trial groups. Initial abstraction was performed by 3 of us (A.D.Z., J.P., and N.S.D.).

### Statistical Analysis

We used descriptive statistics to characterize the sample of new drugs and biologics and their indications for use and to characterize the features of their supporting pivotal trials. We used χ^2^ tests for trend to compare trial characteristics across the 3 periods and Spearman rank tests to compare trial characteristics between the 1995 to 1997 and the 2015 to 2017 periods, both at the level of individual pivotal trials and aggregate pivotal trial evidence supporting each indication approval. To prevent undercounting in cases with missing data, analyses aggregated by indication included only those indications for which pivotal trial characteristic reporting was complete. We stratified analyses by use of any special regulatory program, as defined earlier, and by use of orphan designation.

We conducted sensitivity analyses for individual pivotal trials and aggregated indication approval characteristics to evaluate the association of additional covariates with the observed changes in clinical trial characteristics over time, including product type (small-molecule drug vs biologic), therapeutic area, and expected length of treatment. Expected length of treatment was categorized as acute for drugs with expected lengths of use of less than 1 month, intermediate for those between 1 month and 2 years, and chronic for those longer than 2 years. We also conducted sensitivity analyses examining the individual regulatory programs spanning 3 periods (eg, Priority Review and Accelerated Approval). All statistical tests were 2-tailed and used the Bonferroni method to adjust *P* values to account for multiple comparisons. Statistical significance was set at *P* < .025. All analyses were conducted using R version 3.5.1 (R Project for Statistical Computing).

## Results

We identified 273 new drugs and biologics approved by the FDA for 339 indications across 3 periods: 107 drugs (39.2%) for 157 indications (46.3%) from 1995 to 1997, 57 (20.9%) drugs for 64 indications (18.9%) from 2005 to 2007, and 109 drugs (39.9%) for 118 indications (34.8%) from 2015 to 2017. Product and indication characteristics differed across these periods (Table 1). The proportion of biologics among new approvals has increased (3 [2.8%] in 1995-1997; 8 [14.0%] in 2005-2007; and 30 [27.5%] in 2015-2017; *P* < .001) as have the proportion of approvals using any special regulatory program (37 [34.6%] in 1995-1997; 33 [57.9%] in 2005-2007; and 70 [64.2%] in 2015-2017; *P* < .001) or orphan designation (20 [12.7%] in 1995-1997; 17 [26.6%] in 2005-2007; and 45 [38.1%] in 2015-2017; *P* < .001). The therapeutic areas associated with new indication approvals have shifted, with the most common therapeutic area being infectious disease from 1995 to 1997 (53 [33.8%]) and cancer from 2015 to 2017 (32 [27.1%]) (Table 1).

**Table 1.** Characteristics of New Drugs and Biologics Approved by the US Food and Drug Administration From 1995 to 1997, 2005 to 2007, and 2015 to 2017

Characteristic	No. (%)			*P* value
	1995-1997 (n = 107)	2005-2007 (n = 57)	2015-2017 (n = 109)	
Indications, median (IQR) [range], No.	1 (1-1) [1-14]	1 (1-1) [1-6]	1 (1-1) [1-2]	.03
Agent type				
Drug	104 (97.2)	49 (86.0)	79 (72.5)	<.001
Biologic	3 (2.8)	8 (14.0)	30 (27.5)	<.001
Special regulatory program				
Any	37 (34.6)	33 (57.9)	70 (64.2)	<.001
Priority Review	36 (33.6)	31 (54.4)	67 (61.5)	<.001
Accelerated Approval	12 (11.2)	12 (21.1)	18 (16.5)	.28
Fast Track	NA	14 (24.6)	39 (35.8)	NA
Breakthrough Therapy	NA	NA	34 (31.2)	NA
FDA-approved indications, No.	157	64	118	NA
Therapeutic area				
Infectious disease	53 (33.8)	16 (25.0)	17 (14.4)	<.001
Cancer	17 (10.8)	11 (17.2)	32 (27.1)	<.001
Cardiovascular, diabetes, and/or lipids	26 (16.6)	10 (15.6)	17 (14.4)	.63
Other	61 (38.9)	27 (42.2)	52 (44.1)	.38
Orphan status	20 (12.7)	17 (26.6)	45 (38.1)	<.001

Abbreviations: IQR, interquartile range; NA, not applicable.

### Features of Individual Pivotal Trials

We identified a total of 795 pivotal trials supporting the new drugs and biologics in our sample: 401 trials (50.4%) from 1995 to 1997, 141 trials (17.7%) from 2005 to 2007, and 253 trials (31.8%) from 2015 to 2017. Of these pivotal trials, most were randomized (Table 2), although randomization decreased from 93.6% (95% CI, 90.7%-95.8%) between 1995 and 1997 to 82.2% (95% CI, 74.9%-88.2%) between 2005 and 2007 and 82.2% (95% CI, 76.9%-86.7%) between 2015 and 2017 (*P* < .001). Likewise, most trials were double-blinded, although double-blinding decreased from 79.4% (95% CI, 75.0%-83.3%) between 1995 and 1997 to 67.4% (95% CI, 58.8%-75.0%) between 2005 and 2007 and 67.6% (95% CI, 61.4%-73.3%) between 2015 and 2017 (*P* < .001). Choice of comparators differed by time period; use of active comparators decreased (44.1% [95% CI, 39.2%-49.2%] in 1995-1997; 34.0% [95% CI, 26.3%-42.5%] in 2005-2007; and 29.2% [23.7%-35.3%] in 2015-2017; *P* < .001), while single-group trials increased (8.5% [95% CI, 5.9%-11.6%] in 1995-1997; 17.7% [11.8%-25.1%] in 2005-2007; and 17.8% [13.3%-23.1%] in 2015-2017; *P* < .001). Sensitivity analyses were conducted examining rates of randomization and blinding only among trials using active or placebo comparators (ie, non–single-group trials) and showed no changes over time (eTable 2 in the Supplement).

**Table 2.** Characteristics of Pivotal Trials Supporting New Drugs and Biologics Approved by the US Food and Drug Administration From 1995 to 1997, 2005 to 2007, and 2015 to 2017, Overall and Stratified by Special Regulatory Program Use and Orphan Designation

Characteristic	Trials, No.	% (95% CI)							
		Randomized	Double-blinded	Comparator			End points		
				Active	Placebo	None	Clinical	Scale	Surrogate
**Overall**									
1995-1997	401	93.6 (90.7-95.8)	79.4 (75.0-83.3)	44.1 (39.2-49.2)	47.4 (42.4-52.4)	8.5 (5.9-11.6)	43.8 (38.8-48.8)	8.0 (5.5-11.1)	48.3 (43.3-53.3)
2005-2007	141	82.2 (74.9-88.2)	67.4 (58.8-75.0)	34.0 (26.3-42.5)	48.2 (39.7-56.8)	17.7 (11.8-25.1)	28.4 (21.1-36.6)	11.3 (6.6-17.8)	60.3 (51.7-68.4)
2015-2017	253	82.2 (76.9-86.7)	67.6 (61.4-73.3)	29.2 (23.7-35.3)	53.0 (46.6-59.2)	17.8 (13.3-23.1)	23.3 (18.3-29.0)	17.4 (12.9-22.6)	59.3 (53.0-65.4)
3-Way *P *value	NA	<.001	<.001	<.001	.17	<.001	<.001	<.001	.004
2-Way *P *value^a^	NA	<.001	<.001	<.001			<.001		
**Special regulatory program**									
Any									
1995-1997	89	80.9 (71.2-88.5)	74.4 (63.6-83.4)	37.6 (27.4-48.8)	43.5 (32.8-54.7)	18.8 (11.1-28.8)	20.0 (12.1-30.1)	5.9 (1.9-13.2)	75.3 (64.7-84.0)
2005-2007	64	75.0 (62.6-85.0)	56.3 (43.3-68.6)	35.9 (24.3-48.9)	39.1 (27.1-52.1)	25.0 (15.0-37.4)	37.5 (25.7-50.5)	7.8 (2.6-17.3)	54.7 (41.7-67.2)
2015-2017	128	67.2 (58.3-75.2)	53.9 (44.9-62.8)	23.4 (16.4-31.7)	43.8 (35.0-52.8)	32.8 (24.8-41.7)	19.5 (13.1-27.5)	13.3 (7.9-20.4)	67.2 (58.3-75.2)
3-Way *P *value	NA	.02	.004	.02	.92	.02	.71	.07	.32
2-Way *P *value^a^	NA	.004	.003	.03			.22		
None									
1995-1997	316	96.1 (93.3-98.0)	80.7 (75.9-84.9)	45.9 (40.3-51.6)	48.4 (42.8-54.1)	5.7 (3.4-8.9)	50.2 (44.5-55.8)	8.6 (5.7-12.2)	41.3 (35.8-46.9)
2005-2007	77	88.3 (79.0-94.5)	76.6 (65.6-85.5)	32.5 (22.2-44.1)	55.8 (44.1-67.2)	11.7 (5.5-21.0)	22.1 (13.4-33.0)	14.3 (7.4-24.1)	64.9 (53.2-75.5)
2015-2017	125	97.6 (93.1-99.5)	81.6 (73.7-88.0)	35.2 (26.9-44.2)	62.4 (53.3-70.9)	2.4 (0.5-6.9)	27.2 (19.6-35.9)	21.6 (14.7-29.8)	51.2 (42.1-60.2)
3-Way *P *value	NA	.92	.96	.02	.007	.38	<.001	<.001	.014
2-Way *P *value^a^	NA	.44	.83	.02			<.001		
**Orphan designation**									
Yes									
1995-1997	36	80.6 (62.5-92.5)	61.3 (42.2-78.2)	25.8 (11.9-44.6)	45.2 (27.3-64.0)	29.0 (14.2-48.0)	48.4 (30.2-66.9)	9.7 (2.0-25.8)	41.9 (24.5-60.9)
2005-2007	24	45.8 (25.6-67.2)	29.2 (12.6-51.1)	12.5 (2.7-32.4)	33.3 (15.6-55.3)	54.2 (32.8-74.4)	37.5 (18.8-59.4)	0.0 (0.0-14.2)	62.5 (40.6-81.2)
2015-2017	63	52.4 (39.4-65.1)	39.7 (27.6-52.8)	6.3 (1.8-15.5)	46.0 (33.4-59.1)	47.6 (34.9-60.6)	19.0 (10.2-30.9)	7.9 (2.6-17.6)	73.0 (60.3-83.4)
3-Way *P *value	NA	.02	.09	.009	.80	.13	.003	.94	.004
2-Way *P *value^a^	NA	.009	.05	.02			.009		
No									
1995-1997	372	94.7 (91.9-96.8)	80.9 (76.5-84.9)	45.7 (40.5-50.9)	47.6 (42.4-52.8)	6.8 (4.4-9.8)	43.4 (38.2-48.6)	7.9 (5.3-11.1)	48.8 (43.6-54.0)
2005-2007	117	89.7 (82.8-94.6)	75.2 (66.4-82.7)	38.5 (29.6-47.9)	51.3 (41.9-60.6)	10.3 (5.4-17.2)	26.5 (18.8-35.5)	13.7 (8.0-21.3)	59.8 (50.4-68.8)
2015-2017	190	92.1 (87.3-95.5)	76.8 (70.2-82.6)	36.8 (30.0-44.1)	55.3 (47.9-62.5)	7.9 (4.5-12.7)	24.7 (18.8-31.5)	20.5 (15.0-27.0)	54.7 (47.4-62.0)
3-Way *P *value	NA	.17	.21	.04	.08	.53	<.001	<.001	.12
2-Way *P *value^a^	NA	.22	.26	.14			<.001		

Abbreviation: NA, not applicable.

^a^ Two-way *P* value was calculated for differences between 1995 to 1997 and 2015 to 2017 periods.

Choice of primary end point also differed over time; use of clinical end points decreased (43.8% [95% CI, 38.8%-48.8%] in 1995-1997; 28.4% [95% CI, 21.1%-36.6%] in 2005-2007; and 23.3% [18.3%-29.0%] in 2015-2017; *P* < .001), while the use of surrogate end points increased (48.3% [95% CI, 43.3%-53.3%] in 1995-1997; 60.3% [51.7%-68.4%] in 2005-2007; and 59.3% [53.0%-65.4%] in 2015-2017; *P* = .004). Median (interquartile range [IQR]) number of treated patients in each pivotal trial also increased (277 [150-442] in 1995-1997; 404 [189-622] in 2005-2007; and 467 [209-722] in 2015-2017; *P* < .001), as did median (IQR) trial duration (11.0 [4.9-24.0] weeks in 1995-1997; 16.0 [6.0-26.0] weeks in 2005-2007; and 24.0 [12.0-37.6] weeks in 2015-2017; *P* < .001) (Table 3).

**Table 3.** Number of Treated Patients and Duration of Pivotal Trials Supporting New Drugs and Biologics Approved by the US Food and Drug Administration From 1995 to 1997, 2005 to 2007, and 2015 to 2017, Overall and Stratified by Special Regulatory Program Use and Orphan Designation

Characteristic	Trials, No.	Treated patients, median (IQR), No.		Duration, median (IQR), wk
		Overall	Intervention	
**Overall**				
1995-1997	401	277 (150-442)	168 (91-274)	11.0 (4.9-24.0)
2005-2007	141	404 (189-622)	243 (136-381)	16.0 (6.0-26.0)
2015-2017	253	467 (209-722)	279 (143-451)	24.0 (12.0-37.6)
3-Way *P *value	NA	<.001	<.001	<.001
2-Way *P *value^a^	NA	<.001	<.001	<.001
**Special regulatory program**				
Any				
1995-1997	89	236 (95-424)	148 (64-259)	24.0 (16.0-52.0)
2005-2007	64	329 (115-605)	225 (106-336)	17.0 (9.7-28.3)
2015-2017	128	239 (121-658)	177 (87-366)	24.0 (12.0-50.3)
3-Way *P *value	NA	.10	.03	.61
2-Way *P *value^a^	NA	.07	.02	.33
None				
1995-1997	316	290 (165-451)	182 (97-289)	8.0 (4.0-16.0)
2005-2007	77	455 (273-651)	254 (163-409)	12.0 (5.0-26.0)
2015-2017	125	546 (427-931)	329 (244-574)	24.0 (12.0-26.0)
3-Way *P *value	NA	<.001	<.001	<.001
2-Way *P *value^a^	NA	<.001	<.001	<.001
**Orphan designation**				
Yes				
1995-1997	36	155 (51-270)	94 (28-199)	24.5 (7.9-78.2)
2005-2007	24	92 (55-190)	76 (42-150)	21.3 (8.5-52.0)
2015-2017	63	129 (69-378)	113 (60-242)	34.6 (14.9-70.7)
3-Way *P *value	NA	.71	.20	.08
2-Way *P *value^a^	NA	.98	.30	.19
No				
1995-1997	372	290 (158-446)	179 (97-285)	8.1 (4.8-24.0)
2005-2007	117	471 (296-652)	265 (173-401)	12.0 (5.2-26.0)
2015-2017	190	543 (352-818)	323 (220-503)	20.0 (12.0-26.0)
3-Way *P *value	NA	<.001	<.001	<.001
2-Way *P *value^a^	NA	<.001	<.001	<.001

Abbreviations: IQR, interquartile range; NA, not applicable.

^a^ Two-way *P* value was calculated for differences between 1995 to 1997 and 2015 to 2017 time periods.

### Features of Aggregated Pivotal Trials Supporting Indication Approvals

Overall, 7 of 339 indication approvals (2.0%) were not supported by any pivotal trial (eTable 3 in the Supplement). Of the 332 indication approvals (98.0%) supported by pivotal trials, 293 (88.3%) had complete reporting of all abstracted pivotal trial characteristics within FDA documentation (124 [82.7%] in 1995-1997; 63 [98.4%] in 2005-2007; and 106 [89.8%] in 2015-2017) (eTable 4 and eTable 5 in the Supplement). Among these 293 indication approvals, the proportion supported by at least 2 pivotal trials decreased over time (80.6% [95% CI, 72.6%-87.2%] in 1995-1997; 60.3% [47.2%-72.4%] in 2005-2007; and 52.8% [42.9%-62.6%] in 2015-2017; *P* < .001) (Table 4). The proportion of indication approvals supported only by single-group trials increased over time (4.0% [1.3%-9.2%] in 1995-1997; 12.7% [5.6%-23.5%] in 2005-2007; and 17.0% [10.4%-25.5%] in 2015-2017; *P* = .001). The proportion of indication approvals supported only by trials using surrogate end points was not statistically different over time nor was the median aggregated number of treated patients (Table 4 and Table 5). The proportion of indication approvals with at least 1 trial of 6 months’ duration increased (25.8% [95% CI, 18.4%-34.4%] in 1995-1997; 34.9% [95% CI, 23.3%-48.0%] in 2005-2007; and 46.2% [95% CI, 36.5%-56.2%] in 2015-2017; *P* = .001).

**Table 4.** Characteristics of Aggregated Pivotal Trials Supporting US Food and Drug Administration Indication Approvals for New Drugs and Biologics From 1995 to 1997, 2005 to 2007, and 2015 to 2017, Overall and Stratified by Special Regulatory Program Use and Orphan Designation

Characteristic	Trials, No.	% (95% CI)						
		≥2 Trials	Duration		Comparators		End points	
			≥1 Trial of ≥6 mo	≥1 Trial of ≥12 mo	≥1 Trial using any comparator	Single-group only	≥1 Trial using clinical end points	Trials using surrogate end points only
**Overall**								
1995-1997	124	80.6 (72.6-87.2)	25.8 (18.4-34.4)	18.5 (12.1-26.5)	96.0 (90.8-98.7)	4.0 (1.3-9.2)	60.5 (51.3-69.1)	39.5 (30.9-48.7)
2005-2007	63	60.3 (47.2-72.4)	34.9 (23.3-48.0)	19.0 (10.2-30.9)	87.3 (76.5-94.4)	12.7 (5.6-23.5)	49.2 (36.4-62.1)	50.8 (37.9-63.6)
2015-2017	106	52.8 (42.9-62.6)	46.2 (36.5-56.2)	35.8 (26.8-45.7)	83.0 (74.5-89.6)	17.0 (10.4-25.5)	54.7 (44.8-64.4)	45.3 (35.6-55.2)
3-Way *P *value	NA	.001	.001	.003	.001		.36	
2-Way *P *value^a^	NA	.001	.001	.003	.001		.38	
**Special regulatory program**								
Any								
1995-1997	32	75.0 (56.6-88.5)	50.0 (31.9-68.1)	31.3 (16.1-50.0)	87.5 (71.0-96.5)	12.5 (3.5-29.0)	34.4 (18.6-53.2)	65.6 (46.8-81.4)
2005-2007	36	52.8 (35.5-69.6)	36.1 (20.8-53.8)	25.0 (12.1-42.2)	77.8 (60.8-89.9)	22.2 (10.1-39.2)	47.2 (30.4-64.5)	52.8 (35.5-69.6)
2015-2017	63	38.1 (26.1-51.2)	50.8 (37.9-63.6)	36.5 (24.7-49.6)	73.0 (60.3-83.4)	27.0 (16.6-39.7)	44.4 (31.9-57.5)	55.6 (42.5-68.1)
3-Way *P *value	NA	.001	.74	.48	.11		.42	
2-Way *P *value^a^	NA	.001	.95	.62	.11		.35	
None								
1995-1997	92	82.7 (73.3-89.7)	17.4 (10.3-26.7)	14.1 (7.7-23.0)	98.9 (94.1-100.0)	1.1 (0.0-5.9)	69.6 (59.1-78.7)	30.4 (21.3-40.9)
2005-2007	27	70.4 (49.8-86.2)	33.3 (16.5-54.0)	11.1 (2.4-29.2)	100.0 (87.2-100.0)	0.0 (0.0-12.8)	51.9 (31.9-71.3)	48.1 (28.7-68.1)
2015-2017	43	74.4 (58.8-86.5)	39.5 (25.0-55.6)	34.9 (21.0-50.9)	97.7 (87.7-99.9)	2.3 (0.0-12.3)	69.8 (53.9-82.8)	30.2 (17.2-46.1)
3-Way *P *value	NA	.22	.004	.008	.62		.80	
2-Way *P *value^a^	NA	.27	.006	.006	.59		.98	
**Orphan designation**								
Yes								
1995-1997	11	72.7 (39.0-94.0)	54.5 (23.4-83.3)	36.4 (10.9-69.2)	81.8 (48.2-97.7)	18.2 (2.3-51.8)	81.8 (48.2-97.7)	18.2 (2.3-51.8)
2005-2007	17	23.5 (6.8-49.9)	52.9 (27.8-77.0)	41.2 (18.4-67.1)	58.8 (32.9-81.6)	41.2 (18.4-67.1)	47.1 (23.0-72.2)	52.9 (27.8-77.0)
2015-2017	38	23.7 (11.4-40.2)	63.2 (46.0-78.2)	44.7 (28.6-61.7)	65.8 (48.6-80.4)	34.2 (19.6-51.4)	36.8 (21.8-54.0)	63.2 (46.0-78.2)
3-Way *P *value	NA	.008	.50	.61	.49		.01	
2-Way *P *value^a^	NA	.003	.62	.63	.32		.10	
No								
1995-1997	113	81.4 (73.0-88.1)	23.0 (15.6-31.9)	16.8 (10.4-25.0)	97.3 (92.4-99.4)	2.7 (0.6-7.6)	58.4 (48.8-67.6)	41.6 (32.4-51.2)
2005-2007	46	73.9 (58.9-85.7)	28.3 (16.0-43.5)	10.9 (3.6-23.6)	97.8 (88.5-99.9)	2.2 (0.0-11.5)	50.0 (34.9-65.1)	50.0 (34.9-65.1)
2015-2017	68	69.1 (56.7-79.8)	36.8 (25.4-49.3)	30.9 (20.2-43.3)	92.6 (83.7-97.6)	7.4 (2.4-16.3)	64.7 (52.2-75.9)	35.3 (24.1-47.8)
3-Way *P *value	NA	.06	.05	.04	.14		.50	
2-Way *P *value^a^	NA	.06	.05	.03	.14		.40	

Abbreviation: NA, not applicable.

^a^ Two-way *P* value was calculated for differences between 1995 to 1997 and 2015 to 2017 time periods.

**Table 5.** Number of Pivotal Efficacy Trials and Number of Treated Patients in Aggregated Pivotal Trials Supporting US Food and Drug Administration Indication Approvals of New Drugs and Biologics From 1995 to 1997, 2005 to 2007, and 2015 to 2017, Overall and Stratified by Special Regulatory Program Use and Orphan Designation

Characteristic	Trials, No.	Pivotal efficacy trials, median (IQR), No.	Treated patients, median (IQR), aggregated No.	
			Overall	Intervention
**Overall**				
1995-1997	124	2.0 (2.0-3.0)	774 (464-1314)	490 (236-738)
2005-2007	63	2.0 (1.0-2.8)	699 (218-1380)	416 (191-808)
2015-2017	106	1.0 (1.0-3.0)	816 (199-2112)	523 (145-1303)
3-Way *P *value	NA	.001	.89	.80
2-Way *P *value^a^	NA	.001	.83	.73
**Special regulatory program**				
Any				
1995-1997	32	2.0 (1.4-3.0)	618 (223-945)	384 (147-568)
2005-2007	36	2.0 (1.0-2.0)	534 (166-979)	340 (119-544)
2015-2017	63	1.0 (1.0-2.0)	404 (137-1076)	261 (101-710)
3-Way *P *value	NA	.002	.40	.54
2-Way *P *value^a^	NA	.001	.31	.47
None				
1995-1997	92	2.0 (2.0-3.0)	805 (531-1403)	514 (293-970)
2005-2007	27	2.0 (1.0-3.8)	1167 (419-1835)	680 (244-1374)
2015-2017	43	2.0 (1.2-3.0)	1740 (1047-2853)	1050 (565-1768)
3-Way *P *value	NA	.41	.001	.001
2-Way *P *value^a^	NA	.43	.001	.001
**Orphan designation**				
Yes				
1995-1997	11	2.0 (1.2-2.0)	260 (212-893)	153 (137-444)
2005-2007	17	1.0 (1.0-1.3)	164 (73-422)	83 (42-273)
2015-2017	38	1.0 (1.0-1.1)	196 (90-495)	134 (79-326)
3-Way *P *value	NA	.02	.42	.72
2-Way *P *value^a^	NA	.003	.08	.25
No				
1995-1997	113	2.0 (2.0-3.0)	811 (526-1369)	510 (283-791)
2005-2007	46	2.0 (1.0-3.0)	1027 (474-1584)	577 (267-955)
2015-2017	68	2.0 (1.0-3.0)	1646 (768-2668)	1019 (428-1568)
3-Way *P *value	NA	.11	.001	.001
2-Way *P *value^a^	NA	.14	.001	.001

Abbreviations: IQR, interquartile range; NA, not applicable.

^a^ Two-way *P *value was calculated for differences between 1995 to 1997 and 2015 to 2017 periods.

### Aggregated Pivotal Trial Features Supporting Indication Approvals by Use of Special Regulatory Programs

The proportion of new drug or biologic approvals using any special regulatory program (Priority Review, Accelerated Approval, Fast Track, or Breakthrough Therapy) increased over time, with 37 (34.6%) from 1995 to 1997, 33 (57.9%) from 2005 to 2007, and 70 (64.2%) from 2015 to 2017 (*P* < .001) (Table 1). Among indication approvals using any special regulatory program, the proportion supported by at least 2 pivotal trials decreased over time (75.0% [95% CI, 56.6%-88.5%] in 1995-1997; 52.8% [95% CI, 35.5%-69.6%] in 2005-2007; and 38.1% [95% CI, 26.1%-51.2%] in 2015-2017; *P* < .001) (Table 4). There were not statistically significant changes in the proportion supported only by single-group trials, supported only by trials using surrogate end points, supported by at least 1 trial of 6 months’ duration, or in the median aggregated number of treated patients (Table 4 and Table 5).

Among indication approvals not using any special regulatory program, there were not statistically significant changes in the proportion supported by at least 2 pivotal trials, supported only by single-group trials, or supported by trials using surrogate end points (Table 4). However, the median (IQR) aggregated number of treated patients increased over time (805 [531-1403] in 1995-1997; 1167 [419-1835] in 2005-2007; and 1740 [1047-2853] in 2015-2017; *P* < .001), as did the proportion supported by at least 1 trial of 6 months’ duration (17.4% [95% CI, 10.3%-26.7%] in 1995-1997; 33.3% [95% CI, 16.5%-54.0%] in 2005-2007; and 39.5% [25.0%-55.6%] in 2015-2017; *P* = .004) (Table 4 and Table 5).

### Aggregated Pivotal Trial Features Supporting Indication Approvals by Orphan Designation Status

The proportion of indication approvals receiving orphan designations increased over time, with 20 (12.7%) in 1995 to 1997, 17 (26.6%) in 2005 to 2007, and 45 (38.1%) in 2015 to 2017 (*P* < .001) (Table 1). Trends in the features of aggregated pivotal trials supporting indications over time differed when stratified by orphan designation status. Among orphan indications, the proportion of indication approvals supported by at least 2 pivotal trials decreased over time (72.7% [95% CI, 39.0%-94.0%] in 1995-1997; 23.5% [6.8%-49.9%] in 2005-2007; and 23.7% [11.4%-40.2%] in 2015-2017; *P* = .008) (Table 4). The proportion of orphan indications supported only by single-group trials did not change, while those supported only by trials using surrogate end points increased over time (18.2% [95% CI, 2.3%-51.8%] in 1995-1997; 52.9% [95% CI, 27.8%-77.0%] in 2005-2007; and 63.2% [95% CI, 46.0%-78.2%] in 2015-2017; *P* = .01). The median aggregated number of treated patients for orphan indications did not differ over time nor did the proportion of indications with at least 1 trial of 6 months’ duration (Table 4 and Table 5).

In contrast, the proportion of nonorphan indications supported by at least 2 pivotal trials did not change over time nor did those supported only by single-group trials, those supported only by trials using surrogate end points, or those supported by at least 1 trial of 6 months’ duration. Meanwhile, the median (IQR) aggregated number of treated patients increased (811 [526-1369] in 1995-1997; 1027 [474-1584] in 2005-2007; and 1646 [768-2668] in 2015-2017; *P* < .001).

### Sensitivity Analyses

Observed changes in individual pivotal trial and aggregated indication approval characteristics when stratified by product type were consistent for small-molecule drugs and limited by small sample size for biologics. Observed changes when stratified by therapeutic area were largely consistent, although often limited by small sample size after stratification (eTables 6-9 in the Supplement). When stratified by expected length of treatment, the observed trend of increasing clinical trial duration over time persisted for therapeutics with acute length but not for intermediate or chronic length of treatment (eTable 10 and eTable 11 in the Supplement). The observed trends in aggregated indication approval characteristics were consistent when stratified by Priority Review and Accelerated Approval considered individually (eTable 12 and eTable 13 in the Supplement) compared with special regulatory programs considered as a whole, as were trends when considering orphan designation as a special regulatory program (eTable 14 and eTable 15 in the Supplement).

## Discussion

We reviewed all new drugs and biologics approved by the FDA from 1995 to 1997, from 2005 to 2007, and from 2015 to 2017 and found differences over time in the quality of evidence supporting their approval. The aggregated evidence supporting indication approvals has become less rigorous in some ways, with the proportion of approvals supported by the commonly understood standard of at least 2 pivotal trials declining from 81% to 53% and the proportion of approvals supported by at least 1 trial using a comparator declining from 96% to 83%. Meanwhile, it has become more rigorous in other ways, with the proportion of indications supported by at least 1 trial of 6 months’ duration increasing from 26% to 46%. These findings have implications for patients and clinicians making decisions about whether to use products newly available on the market as well as clarifying the need for continued evaluation of the safety and efficacy of therapeutics after approval.

A key question is whether these overall trends are driven by changes in the baseline characteristics of drug approvals (eg, the frequency with which approvals use special regulatory programs or orphan designation) or whether there has been an evolution in the standards needed to secure approval within these strata. Our findings suggest both explanations may play a role. We found a significant increase in the proportion of new indications approved using any special regulatory program or orphan designation, in line with previous reports.^7^ These changes likely contribute to the trends we observed in clinical trial evidence supporting approvals, given that the use of special regulatory programs and orphan designation are both associated with more flexible standards for approval.^5,19^ However, we also found changes in the aggregated evidence over time even when accounting for use of these programs, suggesting potential involvement of factors beyond compositional effects. There was a decrease in the number of pivotal trials supporting an indication approval only among therapeutics using a special regulatory program, while therapeutics not using a special regulatory program showed increases in the aggregated number of treated patients and the proportion of indications supported by a trial of at least 6 months’ duration. These trends were consistent when examining individual associations with Priority Review, Accelerated Approval, and orphan designation, which were used in all 3 periods studied.

These divergent patterns in evidentiary requirements highlight the trade-offs in premarket evidence development inherent to special regulatory programs and orphan status. These programs are intended to encourage development in areas of particular unmet need and, accordingly, to facilitate approvals based on fewer trials, shorter trials, or trials using surrogate markers, thus requiring less time to ascertain an effect and enabling products to reach the market sooner. Yet the use of these programs has especially proliferated in the new development paradigm of precision medicine, which is defined by narrow target populations, some applications of which may fall outside conventional definitions of unmet need.^20^ Given the corresponding trade-offs in premarket evidence, this has led to increased scrutiny regarding whether the programs’ flexibility in standards are being appropriately applied, especially in cases in which therapeutics are eventually used for indications much broader than those initially approved.^21,22,23^

These issues may continue to intensify as the pipeline for precision medicine matures, and they also highlight the importance of a life cycle approach to evaluating drug efficacy and safety in today’s regulatory environment.^24^ Prior studies^25,26^ have shown that the FDA is more likely to take postmarket safety actions, such as issuing a black box warning or safety communication, for drugs and biologics that received Accelerated Approval or underwent Priority Review. In the context of more flexible premarket standards for evaluation, proponents of this approach embrace the use of postmarketing studies,^8^ pragmatic trials, and real-world evidence (RWE) to ensure continued evidence development for new medical products. In part spurred by the 21st Century Cures Act, the FDA has particularly embraced the use of RWE, defined expansively to include clinical evidence from a variety of settings, including electronic health records, insurance claims, registries, and personal devices, as a new frontier of regulatory science.^27^ In the past few years, the FDA has furthered the integration of RWE into its medical product evaluation process, issuing guidance on the use of RWE in decision-making about drugs, biologics, and devices.^28,29^ Continued development of life cycle evaluation methods, including enhanced requirements that ensure studies are undertaken and reported, may strengthen efforts to generate clinical evidence on the safety and efficacy of drugs and biologics after approval to inform patients, clinicians, and regulators.

### Limitations

Our study has several limitations. First, our study included only 3-year samples of approvals in each period and cannot capture the full range of therapeutic agents and variations in approval trends across entire decades. Secular trends influenced the number of approvals in any given year, and more reduced sample sizes in certain years also influenced the certainty in our evaluation of trends over time. Second, given our sample, we could not fully adjust for associations between all combinations of drug attributes and special regulatory programs and orphan status. For instance, it was not uncommon for a therapeutic approval to use multiple special regulatory programs, and we could not fully account for the possibility that a single regulatory program or attribute had disproportionate influence on the associations observed. However, the consistency of our findings regarding special regulatory programs across multiple individual special regulatory programs as well as orphan designation suggests that we were able to capture major trends. Third, we included only clinical trials identified as pivotal trials in our study. Other nonpivotal studies and data, such as observational studies or previous marketing experience from other countries, may contribute to FDA reviewers’ holistic evaluations of drugs under consideration in ways that cannot be captured by our approach.

## Conclusions

The findings of this study suggest that quality of clinical trial evidence used to support new drug and biologic approvals has changed during the past 3 decades, requiring fewer pivotal trials with less robust comparators but with longer durations. This change has implications for physicians and patients as they consider using newly approved drugs as well as for regulators, given that it suggests an increasing need for continued evaluation of therapeutic safety and efficacy after approval.

## Notes

Supplement. **eAppendix. **Details on the Characterization of Pivotal Trials Supporting New Drugs and Biologics Approved by the US Food and Drug Administration **eTable 1.** Special Regulatory Program Characteristics **eTable 2. **Randomization and Blinding of Pivotal Trials Supporting New Drugs and Biologics Approved by the US Food and Drug Administration in 1995-1997, 2005-2007, and 2015-2017, Overall and Limited to Trials with Comparator Arms **eTable 3.** New Drugs and Biologics Approved by the US Food and Drug Administration in 1995-1997, 2005-2007, and 2015-2017 Without Pivotal Efficacy Trials **eTable 4. **Availability of Information Within Requested Documents for Specific Pivotal Trial Characteristics for New Drugs and Biologics Approved by the US Food and Drug Administration in 1995-1997, Overall and Stratified by Special Regulatory Program and Orphan Designation **eTable 5. **Availability of Information for Specific Aggregated Pivotal Trial Characteristics for New Drug and Biologic Indications Approved by the US Food and Drug Administration in 1995-1997, 2005-2007, and 2015-2017, Overall and Stratified by Approval Year **eTable 6. **Characteristics of Pivotal Trials Supporting New Drugs and Biologics Approved by the US Food and Drug Administration in 1995-1997, 2005-2007, and 2015-2017, Stratified by Drug Type **eTable 7. **Characteristics of Aggregated Pivotal Trials Supporting New Drug and Biologic Indications Approved by the US Food and Drug Administration in 1995-1997, 2005-2007, and 2015-2017, Stratified by Drug Type **eTable 8.** Characteristics of Pivotal Trials Supporting New Drugs and Biologics Approved by the US Food and Drug Administration in 1995-1997, 2005-2007, and 2015-2017, Stratified by Therapeutic Area **eTable 9. **Characteristics of Aggregated Pivotal Trials Supporting New Drug and Biologic Indications Approved by the US Food and Drug Administration in 1995-1997, 2005-2007, and 2015-2017, Stratified by Therapeutic Area **eTable 10. **Median Duration of Pivotal Trials Supporting New Drugs and Biologics Approved by the US Food and Drug Administration in 1995-1997, 2005-2007, and 2015-2017, Overall and Stratified by Expected Length of Treatment **eTable 11. **Duration of Aggregated Pivotal Trials Supporting New Drug and Biologic Indications Approved by the US Food and Drug Administration in 1995-1997, 2005-2007, and 2015-2017, Overall and Stratified by Expected Length of Treatment **eTable 12. **Characteristics of Pivotal Trials Supporting New Drugs and Biologics Approved by the US Food and Drug Administration in 1995-1997, 2005-2007, and 2015-2017, Stratified by Use of Priority Review and Accelerated Approval **eTable 13. **Characteristics of Aggregated Pivotal Trials Supporting New Drugs and Biologic Indications Approved by the US Food and Drug Administration in 1995-1997, 2005-2007, and 2015-2017, Stratified by Use of Priority Review and Accelerated Approval **eTable 14. **Characteristics of Pivotal Trials Supporting New Drugs and Biologics Approved by the US Food and Drug Administration in 1995-1997, 2005-2007, and 2015-2017, Stratified by Use of Any Special Regulatory Program, Considering Orphan Designation as a Special Regulatory Program **eTable 15. **Characteristics of Aggregated Pivotal Trials Supporting New Drugs and Biologic Indications Approved by the US Food and Drug Administration in 1995-1997, 2005-2007, and 2015-2017, Stratified by Use of Any Special Regulatory Program, Considering Orphan Designation as a Special Regulatory Program
