# Supplementary material for: Assessment of Clinical Trials Supporting US Food and Drug Administration Approval of Novel Therapeutic Agents, 1995-2017
Source: JAMA Netw Open. 2020 Apr 21;3(4):e203284. doi: 10.1001/jamanetworkopen.2020.3284 (PMC7175081; doi:10.1001/jamanetworkopen.2020.3284)
Supplement: Supplement. — eAppendix. Details on the Characterization of Pivotal Trials Supporting New Drugs and Biologics Approved by the US Food and Drug Administration eTable 1. Special Regulatory Program Characteristics eTable 2. Randomization and Blinding of Pivotal Trials Supporting New Drugs and Biologics Approved by the US Food and Drug Administration in 1995-1997, 2005-2007, and 2015-2017, Overall and Limited to Trials with Comparator Arms eTable 3. New Drugs and Biologics Approved by the US Food and Drug Administration in 1995-1997, 2005-2007, and 2015-2017 Without Pivotal Efficacy Trials eTable 4. Availability of Information Within Requested Documents for Specific Pivotal Trial Characteristics for New Drugs and Biologics Approved by the US Food and Drug Administration in 1995-1997, Overall and Stratified by Special Regulatory Program and Orphan Designation eTable 5. Availability of Information for Specific Aggregated Pivotal Trial Characteristics for New Drug and Biologic Indications Approved by the US Food and Drug Administration in 1995-1997, 2005-2007, and 2015-2017, Overall and Stratified by Approval Year eTable 6. Characteristics of Pivotal Trials Supporting New Drugs and Biologics Approved by the US Food and Drug Administration in 1995-1997, 2005-2007, and 2015-2017, Stratified by Drug Type eTable 7. Characteristics of Aggregated Pivotal Trials Supporting New Drug and Biologic Indications Approved by the US Food and Drug Administration in 1995-1997, 2005-2007, and 2015-2017, Stratified by Drug Type eTable 8. Characteristics of Pivotal Trials Supporting New Drugs and Biologics Approved by the US Food and Drug Administration in 1995-1997, 2005-2007, and 2015-2017, Stratified by Therapeutic Area eTable 9. Characteristics of Aggregated Pivotal Trials Supporting New Drug and Biologic Indications Approved by the US Food and Drug Administration in 1995-1997, 2005-2007, and 2015-2017, Stratified by Therapeutic Area eTable 10. Median Duration of Pivotal Trials Supporting New Drugs and [file jamanetwopen-3-e203284-s001.pdf]

Supplementary Online Content

Zhang AD, Puthumana J, Downing NS, Shah ND, Krumholz HM, Ross JS. Assessment of clinical trials supporting us food and drug administration approval of novel therapeutic agents, 1995-2017. *JAMA Newt Open*. 2020;3(4):e203284. doi:10.1001/jamanetworkopen.2020.3284

eAppendix. Details on the Characterization of Pivotal Trials Supporting New Drugs and Biologics Approved by the US Food and Drug Administration
eTable 1. Special Regulatory Program Characteristics
eTable 2. Randomization and Blinding of Pivotal Trials Supporting New Drugs and Biologics Approved by the US Food and Drug Administration in 1995-1997, 2005-2007, and 2015-2017, Overall and Limited to Trials with Comparator Arms
eTable 3. New Drugs and Biologics Approved by the US Food and Drug Administration in 1995-1997, 2005-2007, and 2015-2017 Without Pivotal Efficacy Trials
eTable 4. Availability of Information Within Requested Documents for Specific Pivotal Trial Characteristics for New Drugs and Biologics Approved by the US Food and Drug Administration in 1995-1997, Overall and Stratified by Special Regulatory Program and Orphan Designation
eTable 5. Availability of Information for Specific Aggregated Pivotal Trial Characteristics for New Drug and Biologic Indications Approved by the US Food and Drug Administration in 1995-1997, 2005-2007, and 2015-2017, Overall and Stratified by Approval Year
eTable 6. Characteristics of Pivotal Trials Supporting New Drugs and Biologics Approved by the US Food and Drug Administration in 1995-1997, 2005-2007, and 2015-2017, Stratified by Drug Type
eTable 7. Characteristics of Aggregated Pivotal Trials Supporting New Drug and Biologic Indications Approved by the US Food and Drug Administration in 1995-1997, 2005-2007, and 2015-2017, Stratified by Drug Type
eTable 8. Characteristics of Pivotal Trials Supporting New Drugs and Biologics Approved by the US Food and Drug Administration in 1995-1997, 2005-2007, and 2015-2017, Stratified by Therapeutic Area
eTable 9. Characteristics of Aggregated Pivotal Trials Supporting New Drug and Biologic Indications Approved by the US Food and Drug Administration in 1995-1997, 2005-2007, and 2015-2017, Stratified by Therapeutic Area
eTable 10. Median Duration of Pivotal Trials Supporting New Drugs and Biologics Approved by the US Food and Drug Administration in 1995-1997, 2005-2007, and 2015-2017, Overall and Stratified by Expected Length of Treatment
eTable 11. Duration of Aggregated Pivotal Trials Supporting New Drug and Biologic Indications Approved by the US Food and Drug Administration in 1995-1997, 2005-2007, and 2015-2017, Overall and Stratified by Expected Length of Treatment

© 2020 Zhang AD et al. *JAMA Network Open.*

eTable 12. Characteristics of Pivotal Trials Supporting New Drugs and Biologics Approved by the US Food and Drug Administration in 1995-1997, 2005-2007, and 2015-2017, Stratified by Use of Priority Review and Accelerated Approval
eTable 13. Characteristics of Aggregated Pivotal Trials Supporting New Drugs and Biologic Indications Approved by the US Food and Drug Administration in 1995-1997, 2005-2007, and 2015-2017, Stratified by Use of Priority Review and Accelerated Approval
eTable 14. Characteristics of Pivotal Trials Supporting New Drugs and Biologics Approved by the US Food and Drug Administration in 1995-1997, 2005-2007, and 2015-2017, Stratified by Use of Any Special Regulatory Program, Considering Orphan Designation as a Special Regulatory Program
eTable 15. Characteristics of Aggregated Pivotal Trials Supporting New Drugs and Biologic Indications Approved by the US Food and Drug Administration in 1995-1997, 2005-2007, and 2015-2017, Stratified by Use of Any Special Regulatory Program, Considering Orphan Designation as a Special Regulatory Program

This supplementary material has been provided by the authors to give readers additional information about their work.

© 2020 Zhang AD et al. *JAMA Network Open.*

eAppendix. Details on the Characterization of Pivotal Trials Supporting New Drugs and Biologics Approved by the US Food and Drug Administration

Characteristics of pivotal trials were categorized according to a previously defined framework (Downing 2014). Definitions are provided below. Pivotal trials were classified based on descriptions of the pivotal trials provided within the FDA medical review.

Randomization
Trials were classified according to the use of randomization.

- "Randomized" trials were explicitly described within the review as "randomized" or described to be randomized between arms.
- All other trials were classified as "non-randomized".

Double-Blinding
Trials were classified according to level of blinding used.

- "Double-blinded" trials were explicitly described within the review as "double-blinded" or described to be blinded to both investigator and patient.
- All other trials were classified as "non-double-blinded", including those trials described as "single-blinded", blinded to patient only, unblinded or "open-label" trials, or single arm trials.

Comparator
Trials were classified according to the type of comparator used to evaluate the study drug.

- Trials with "active" comparators compared the study drug to at least one other drug (including those trials comparing the study drug to both another drug and placebo).
- Trials with "placebo" comparators compared the study drug only to placebo.
- Trials with "no comparator" compared the study drug only to itself (as in multi-arm trials with multiple doses) or were single-arm trials.

Endpoints
Trials were classified according to the primary endpoint used to assess the efficacy of the study drug.

- Trials with a "clinical endpoint" measure patient-reported outcomes, function, or survival. Examples include:
    - Overall survival
    - Clinical cure rate
    - Seizure frequency
- Trials with a "clinical scale" endpoint use rubrics to quantify subjective patient-reported symptoms. Examples include:
    - Change in Hamilton Rating Scale of Depression score
    - Pain improvement from baseline using visual analog scale
    - Improvement in American College of Rheumatology score
- Trials with a "surrogate endpoint" use biomarkers expected to predict clinical benefit. Examples include:
    - Progression-free survival
    - Objective response rate
    - Change in systolic blood pressure
    - Hemoglobin A1c
    - Sustained virologic response

Composite endpoints were classified according to the component which generated the most patient-pertinent information. For instance, a composite endpoint of overall survival (clinical endpoint) and progression-free survival (surrogate endpoint) would be classified as a clinical endpoint.

Intention-to-Treat Population
Trials were classified by the size of the intention-to-treat population, defined as the number of subjects receiving at least one dose of treatment (whether study medication, comparator, or placebo).

© 2020 Zhang AD et al. *JAMA Network Open.*

Trial Duration

Trials were classified by the duration of the trial.

- Endpoint-driven trials (such as those measuring the primary endpoint such as cure or death at pre-specified intervals): described by the length of time in weeks between the time the first dose of the drug was administered and the time that the primary endpoint was measured.
- Event-driven trials (such as those measuring time to outcomes such as death or hospitalization): described by the median duration of treatment. When differences in duration of treatment existed between treatment arms, a weighted average calculation was used to determine a single value for trial duration.

© 2020 Zhang AD et al. *JAMA Network Open.*

eTable 1. Special Regulatory Program Characteristics

Program	Year	Eligibility Criteria	Benefits
Fast Track	1988 (Subpart H)<br>1997 (FDAMA)	• Drug treats a serious condition<br>• Data demonstrate the potential to address unmet medical need<br>OR<br>• Drug designated as qualified infectious disease product	• Actions to expedite development and review, including meetings with FDA during development[1]
Priority Review	1992	• Drug treats a serious condition<br>• Would provide a significant improvement in safety or effectiveness<br>OR<br>• Drug designated as a qualified infectious disease product<br>OR<br>• Submitted with a priority review voucher	• Shorter clock for review of marketing application
Accelerated Approval	1992	• Drug treats a serious condition<br>• Provides a meaningful advantage over available therapies	• Approval based on effect on surrogate endpoint or intermediate clinical endpoint reasonably likely to predict a drug's clinical benefit
Breakthrough Designation	2012	• Drug intended to treat a serious condition<br>• Preliminary clinical evidence suggests the drug may demonstrate substantial improvement on a clinically-significant endpoint over available therapies	• Intensive guidance on efficient drug development, including meetings with FDA during development that may allow for alternative clinical trial designs<br>• Other actions to expedite review

Source: FDA Guidance: Expedited Programs for Serious Conditions—Drugs and Biologics.
FDAMA = Food and Drug Administration Modernization Act
[1] While the statutory language for Fast Track does not specifically provide for flexible clinical trial standards, guidance issued by the FDA regarding the execution of the Fast Track program at this time suggested that a single phase 2 trial may be considered sufficient to support approval.

© 2020 Zhang AD et al. *JAMA Network Open.*

eTable 2. Randomization and Blinding of Pivotal Trials Supporting New Drugs and Biologics Approved by the US Food and Drug Administration in 1995-1997, 2005-2007, and 2015-2017, Overall and Limited to Trials with Comparator Arms

Characteristic	Randomized, % (95% CI)	Double-Blinded, % (95% CI)
**All Pivotal Trials**		
1995-1997 (n=401)	93.6 (90.7-95.8)	79.4 (75.0-83.3)
2005-2007 (n=141)	82.2 (74.9-88.2)	67.4 (59.0-75.0)
2015-2017 (n=253)	82.2 (76.9-86.7)	67.6 (61.4-73.3)
3-way p	P<0.001	P<0.001
**Controlled Trials Only**		
1995-1997 (n=367)	100 (99.0-100)	84.4 (80.2-88.0)
2005-2007 (n=116)	100 (96.9-100)	81.9 (73.7-88.4)
2015-2017 (n=208)	100 (98.2-100)	82.2 (76.3-87.2)
3-way p	NA	p=0.47

© 2020 Zhang AD et al. *JAMA Network Open.*

eTable 3. New Drugs and Biologics Approved by the US Food and Drug Administration in 1995-1997, 2005-2007, and 2015-2017 Without Pivotal Efficacy Trials

4 drugs in the sample were approved without pivotal efficacy trials, representing 7 indications.

**Drug**	**Year**	**Indications Without Pivotal Efficacy Trials**
Cerbyx (fosphenytoin sodium)	1996	1 - substituted, as short-term use, for oral phenytoin<br>2 - generalized tonic-clonic status epilepticus<br>3 - prevention and treatment of seizures occurring during neurosurgery
Albenza (albendazole)	1996	1 - neurocysticercosis<br>2 - hydatid disease
Cystadine (betaine hydrochloride)	1996	1 - decrease elevated homocysteine blood levels in patients with cystathionine beta-synthase type of homocystinuria; MTHFR deficiency; cobalamin defect
Sclerosol (talc)	1997	1 - treatment of malignant pleural effusions

© 2020 Zhang AD et al. *JAMA Network Open.*

eTable 4. Availability of Information Within Requested Documents for Specific Pivotal Trial Characteristics for New Drugs and Biologics Approved by the US Food and Drug Administration in 1995-1997, Overall and Stratified by Special Regulatory Program and Orphan Designation

**Characteristic**	**Use of Randomization, No. (%)**	**Use of Double-Blinding, No. (%)**	**Choice of Comparator, No. (%)**	**Choice of Endpoint, No. (%)**	**Overall Treated Patients, No. (%)**	**Intervention Treated Patients, No. (%)**	**Duration, No. (%)**
Overall (n=401)	392 (97.8)	393 (98.0)	401 (100)	400 (99.8)	396 (98.8)	391 (97.5)	330 (82.3)
**Special Regulatory Program**							
Any (n=85)	85 (100)	82 (96.5)	85 (100)	85 (100)	85 (100)	85 (100)	68 (80.0)
None (n=316)	307 (97.2)	311 (98.4)	316 (100)	315 (99.7)	311 (98.4)	306 (96.8)	266 (84.2)
**Orphan**							
Yes (n=31)	31 (100)	31 (100)	31 (100)	31 (100)	31 (100)	31 (100)	18 (58.0)
No (n=370)	361 (97.6)	362 (97.8)	370 (100)	369 (99.7)	365 (98.6)	360 (97.3)	311 (84.1)

© 2020 Zhang AD et al. *JAMA Network Open.*

eTable 5. Availability of Information for Specific Aggregated Pivotal Trial Characteristics for New Drug and Biologic Indications Approved by the US Food and Drug Administration in 1995-1997, 2005-2007, and 2015-2017, Overall and Stratified by Approval Year

**Characteristic**	**Choice of Comparator, No. (%)**	**Choice of Endpoint, No. (%)**	**Overall Treated Patients, No. (%)**	**Intervention Treated Patients, No. (%)**	**Duration, No. (%)**	**All Variables, No. (%)**
Overall (n=332)	332 (100)	331 (99.7)	330 (99.4)	327 (98.5)	293 (88.3)	293 (88.3)
1995-1997 (n=150)	150 (100)	149 (99.3)	148 (98.7)	145 (96.7)	124 (82.7)	124 (82.7)
2005-2007 (n=64)	64 (100)	64 (100)	64 (100)	64 (100)	63 (98.4)	63 (98.4)
2015-2017 (n=118)	118 (100)	118 (100)	118 (100)	118 (100)	106 (89.8)	106 (89.8)

© 2020 Zhang AD et al. *JAMA Network Open.*

eTable 6. Characteristics of Pivotal Trials Supporting New Drugs and Biologics Approved by the US Food and Drug Administration in 1995-1997, 2005-2007, and 2015-2017, Stratified by Drug Type

	Random-ized, % (95% CI)	Double-Blinded, % (95% CI)	Comparator, % (95% CI)			Endpoint, % (95% CI)			Median Patients (IQR)		Median Duration (IQR), weeks
			Active	Placebo	None	Clinical	Scale	Surrogate	Overall	Intervention	
**Drug Type**											
Molecule											
1995-1997 (n=396)	93.7 (90.9-96.0)	79.3 (75.0-83.3)	44.7 (39.7-49.7)	47.0 (42.0-52.0)	8.3 (5.8-11.5)	43.5 (38.6-48.6)	7.8 (5.4-11.0)	48.6 (43.6-53.7)	278 (150-443)	170 (92-277)	9.5 (4.9-24.0)
2005-2007 (n=125)	80.8 (72.8-87.3)	69.6 (60.7-77.5)	32.8 (24.7-41.8)	48.0 (39.0-57.1)	19.2 (12.7-27.2)	29.6 (21.8-38.4)	8.8 (4.5-15.2)	61.6 (52.5-70.2)	408 (192-627)	251 (138-385)	12.0 (5.0-24.0)
2015-2017 (n=185)	81.6 (75.3-86.9)	64.9 (57.5-71.7)	29.7 (23.2-36.9)	51.9 (44.4-59.3)	18.4 (13.1-24.7)	22.2 (16.4-28.8)	14.1 (9.4-19.9)	63.8 (56.4-70.7)	464 (217-705)	279 (153-436)	24.0 (12.0-27.4)
3-way p	P<0.001	P<0.001	P<0.001	P=0.23	P<0.001	P<0.001	P=0.02	P<0.001	P<0.001	P<0.001	P<0.001
2-way p	P<0.001	P<0.001	P<0.001			P<0.001			P<0.001	P<0.001	P<0.001
Biologic											
1995-1997 (n=5)	80.0 (28.4-99.5)	80.0 (28.4-99.5)	0.0 (0.0-5.2)	80.0 (28.4-99.5)	20.0 (0.5-71.6)	60.0 (14.7-94.7)	20.0 (0.5-71.6)	20.0 (0.5-71.6)	172 (150-328)	158 (52-196)	20.0 (20.0-39.3)
2005-2007 (n=16)	93.8 (69.8-99.8)	50.0 (24.7-75.3)	43.8 (19.8-70.1)	50.0 (24.7-75.3)	6.3 (0.2-30.2)	18.8 (4.0-45.6)	31.3 (11.0-58.7)	50.0 (24.7-75.3)	338 (131-525)	194 (94-338)	36.0 (24.9-52.0)
2015-2017 (n=68)	83.8 (72.9-91.6)	75.0 (63.0-84.7)	27.9 (17.7-40.1)	55.9 (43.3-67.9)	16.2 (8.4-27.1)	26.5 (16.5-38.6)	26.5 (16.5-38.6)	47.1 (34.8-60.0)	487 (126-835)	308 (86-488)	24.0 (12.0-46.8)
3-way p	P=0.67	P=0.32	P=0.81	P=0.60	P=0.67	P=0.40	P=1.00	P=0.46	P=0.09	P=0.07	P=0.13
2-way p	P=0.84	P=0.82	P=0.38			P=0.27			P=0.18	P=0.14	P=1.00

© 2020 Zhang AD et al. *JAMA Network Open.*

eTable 7. Characteristics of Aggregated Pivotal Trials Supporting New Drug and Biologic Indications Approved by the US Food and Drug Administration in 1995-1997, 2005-2007, and 2015-2017, Stratified by Drug Type

	≥2 Trials (%, 95% CI)	Comparators (%, 95% CI)		Endpoints (%, 95% CI)		Median Patients in Aggregated Pivotal Efficacy Trials (IQR)		Duration (%, 95% CI)	
		≥1 Trial Using Any Comparator	Single-Arm Only	≥1 Trial Using Clinical Endpoints or Clinical Scales	Surrogate Endpoints Only	Overall	Intervention	≥1 Trial of ≥6 Months	≥1 Trial of ≥12 Months
**Drug Type**									
Molecule									
1995-1997 (n=122)	81.1 (73.1-87.7)	95.9 (90.7-98.7)	4.1 (1.3-9.3)	59.8 (50.6-68.6)	40.2 (31.4-49.4)	786 (472-1355)	493 (237-745)	25.4 (18.0-34.1)	18.0 (11.7-26.0)
2005-2007 (n=55)	63.6 (49.6-76.2)	87.3 (75.5-94.7)	12.7 (5.3-24.5)	47.3 (33.7-61.2)	52.7 (38.8-66.3)	707 (301-1433)	425 (196-808)	29.1 (17.6-42.9)	14.5 (6.5-26.7)
2015-2017 (n=79)	48.1 (36.7-59.6)	84.8 (75.0-91.9)	15.2 (8.1-25.0)	51.9 (40.4-63.3)	48.1 (36.7-59.6)	796 (220-1820)	445 (200-1092)	44.3 (33.1-55.9)	35.4 (25.0-47.0)
3-way p	P<0.001	P=0.007		P=0.22		P=0.90	P=0.98	P=0.006	P=0.007
2-way p	P<0.001	P=0.006		P=0.27		P=0.99	P=0.90	P=0.005	P=0.005
Biologic									
1995-1997 (n=2)	50.0 (1.3-98.7)	100 (15.8-100)	0 (0.0-84.2)	100 (15.8-100)	0 (0.0-84.2)	487 (301-672)	255 (158-351)	50.0 (1.3-98.7)	50.0 (1.3-98.7)
2005-2007 (n=8)	37.5 (8.5-75.5)	87.5 (47.3-99.7)	12.5 (0.3-52.7)	62.5 (24.5-91.5)	37.5 (8.5-75.5)	279 (59-1281)	147 (29-847)	75.0 (34.9-96.8)	50.0 (15.7-84.3)
2015-2017 (n=27)	66.7 (46.0-83.5)	77.8 (57.7-91.4)	22.2 (8.6-42.3)	63.0 (42.4-80.6)	37.0 (19.4-57.6)	1327 (94-2448)	825 (85-1343)	51.9 (31.9-71.3)	37.0 (19.4-57.6)
3-way p	P=0.21	P=0.35		P=0.46		P=0.20	P=0.12	P=0.49	P=0.51
2-way p	P=0.68	P=0.50		P=0.32		P=0.70	P=0.60	P=1.00	P=0.76

© 2020 Zhang AD et al. *JAMA Network Open.*

eTable 8. Characteristics of Pivotal Trials Supporting New Drugs and Biologics Approved by the US Food and Drug Administration in 1995-1997, 2005-2007, and 2015-2017, Stratified by Therapeutic Area

	Random-ized, % (95% CI)	Double-Blinded, % (95% CI)	Comparator, % (95% CI)			Endpoint, % (95% CI)			Median Patients (IQR)		Median Duration (IQR), weeks
			Active	Placebo	None	Clinical	Scale	Surrogate	Overall	Intervention	
**Area**											
ID											
1995-1997 (n=102)	96.0 (90.2-98.9)	72.0 (62.1-80.5)	81.4 (72.4-88.4)	14.7 (8.5-23.1)	3.9 (1.1-9.7)	67.3 (57.3-76.3)	0	32.7 (23.7-42.7)	362 (254-553)	200 (144-294)	5.0 (3.4-7.6)
2005-2007 (n=29)	96.6 (82.2-99.9)	75.9 (56.5-89.7)	82.8 (64.2-94.2)	13.8 (3.9-31.7)	3.4 (0.1-17.8)	51.7 (32.5-70.6)	0	48.3 (29.4-67.5)	543 (339-662)	292 (234-385)	16.0 (2.3-24.0)
2015-2017 (n=40)	65.0 (48.3-79.4)	52.5 (36.1-68.5)	32.5 (18.6-49.1)	32.5 (18.6-49.1)	35.0 (20.6-51.7)	27.5 (14.6-43.9)	0	72.5 (56.1-85.4)	405 (167-549)	240 (129-409)	12.0 (11.5-12.0)
3-way p	P<0.001	P=0.05	P<0.001	P=0.02	P<0.001	P<0.001	NA	P<0.001	P=0.56	P=0.07	P=0.001
2-way p	P<0.001	P=0.03	P<0.001			P<0.001			P=0.72	P=0.36	P<0.001
Cancer											
1995-1997 (n=31)	67.7 (48.6-83.3)	39.3 (21.5-59.4)	38.7 (21.8-57.8)	16.1 (5.5-33.7)	45.2 (27.3-64.0)	22.6 (9.6-41.1)	0	77.4 (58.9-90.4)	235 (112-410)	122 (71-260)	26.0 (9.2-56.4)
2005-2007 (n=18)	38.9 (17.3-64.3)	11.1 (1.4-34.7)	11.1 (1.4-34.7)	27.8 (9.7-53.5)	5.6 (0.1-27.3)	5.6 (0.1-27.3)	0	94.4 (72.7-99.9)	139 (74-395)	139 (74-209)	17.0 (5.0-24.5)
2015-2017 (n=40)	52.5 (36.1-68.5)	35.0 (20.6-51.7)	5.0 (0.6-16.9)	47.5 (31.5-63.9)	47.5 (31.5-63.9)	20.0 (9.1-35.6)	0	80.0 (64.4-90.9)	228 (135-598)	215 (124-349)	56.8 (31.5-87.0)
3-way p	P=0.23	P=0.84	P<0.001	P=0.005	P=0.90	P=0.85	NA	P=0.85	P=0.10	P=0.04	P=0.003
2-way p	P=0.20	P=0.73	P<0.001			P=1.00			P=0.14	P=0.05	P=0.06
Cardiovascular											
1995-1997 (n=113)	99.1 (95.1-100)	87.5 (79.9-93.0)	30.1 (21.8-39.4)	69.0 (59.6-77.4)	0.9 (0.0-4.8)	8.8 (4.3-15.7)	0	91.2 (84.3-95.7)	252 (152-442)	155 (86-327)	12.0 (8.0-25.7)
2005-2007 (n=33)	90.9 (75.7-98.1)	84.8 (68.1-94.9)	18.2 (7.0-35.5)	72.7 (54.5-87.6)	9.1 (1.9-24.3)	9.1 (1.9-24.3)	0	90.9 (75.7-98.1)	538 (337-688)	387 (241-499)	12.0 (10.7-26.0)
2015-2017 (n=67)	97.0 (89.6-99.6)	68.7 (56.1-79.4)	53.7 (41.1-66.0)	43.3 (31.2-56.0)	3.0 (0.4-10.4)	14.9 (7.4-25.7)	0	85.1 (74.3-92.6)	484 (365-1020)	313 (221-697)	26.0 (24.0-26.3)
3-way p	P=0.29	P=0.002	P=0.003	P=0.001	P=0.29	P=0.22	NA	P=0.22	P<0.001	P<0.001	P<0.001
2-way p	P=0.30	P=0.002	P=0.003			P=0.31			P<0.001	P<0.001	P<0.001
Other											
1995-1997 (n=155)	93.2 (87.9-96.7)	85.6 (79.0-90.8)	31.0 (23.8-38.9)	59.4 (51.2-67.2)	9.7 (5.5-15.5)	58.1 (49.9-65.9)	20.6 (14.6-27.9)	21.3 (15.1-28.6)	247 (127-375)	155 (77-250)	12.0 (6.0-24.0)
2005-2007 (n=61)	83.6 (71.9-91.8)	70.5 (57.4-81.5)	26.2 (15.8-39.1)	57.4 (44.1-70.0)	16.4 (8.2-28.1)	34.4 (22.7-47.7)	26.2 (15.8-39.1)	39.3 (27.0-52.7)	324 (99-522)	181 (83-302)	16.0 (6.0-36.0)

© 2020 Zhang AD et al. *JAMA Network Open.*

2015-2017 (n=106)	89.6 (82.2-94.7)	84.9 (76.6-91.1)	21.7 (14.3-30.8)	68.9 (59.1-77.5)	9.4 (4.6-16.7)	28.3 (20.0-37.9)	41.5 (32.0-51.5)	30.2 (21.7-39.9)	544 (195-741)	299 (100-473)	13.4 (12.0-25.2)
3-way p	P=0.39	P=0.71	P=0.10	P=0.14	P=0.95	P<0.001	P<0.001	P=0.08	P<0.001	P<0.001	P=0.06
2-way p	P=0.44	P=0.87	P=0.24			P<0.001			P<0.001	P<0.001	P=0.05

© 2020 Zhang AD et al. *JAMA Network Open.*

eTable 9. Characteristics of Aggregated Pivotal Trials Supporting New Drug and Biologic Indications Approved by the US Food and Drug Administration in 1995-1997, 2005-2007, and 2015-2017, Stratified by Therapeutic Area

	**≥2 Trials (%, 95% CI)**	**Comparators (%, 95% CI)**		**Endpoints (%, 95% CI)**		**Median Patients in Aggregated Pivotal Efficacy Trials (IQR)**		**Duration (%, 95% CI)**	
		**≥1 Trial Using Any Comparator**	**Single-Arm Only**	**≥1 Trial Using Clinical-Type Endpoints**	**Surrogate Endpoints Only**	**Overall**	**Intervention**	**≥1 Trial of ≥6 Months**	**≥1 Trial of ≥12 Months**
**Area**									
ID									
1995-1997 (n=48)	68.8 (53.7-81.3)	100 (92.6-100)	0 (0.0-7.4)	72.9 (58.2-84.7)	27.1 (15.3-41.8)	761 (464-1106)	436 (236-659)	4.2 (0.5-14.3)	2.1 (0.0-11.1)
2005-2007 (n=16)	68.8 (41.3-89.0)	100 (79.4-100)	0 (0.0-20.6)	56.3 (29.9-80.2)	43.8 (19.8-70.1)	998 (663-1285)	540 (433-777)	12.5 (1.6-38.3)	6.3 (0.2-30.2)
2015-2017 (n=16)	62.5 (35.4-84.8)	93.8 (69.8-99.8)	6.3 (0.2-30.2)	50.0 (24.7-75.3)	50.0 (24.7-75.3)	647 (305-1644)	344 (177-1157)	12.5 (1.6-38.3)	6.3 (0.2-30.2)
3-way p	P=0.68	P=0.08		P=0.07		P=0.72	P=0.59	P=0.20	P=0.37
2-way p	P=0.65	P=0.09		P=0.10		P=0.84	P=1.00	P=0.24	P=0.43
Cancer									
1995-1997 (n=7)	57.1 (18.4-90.1)	71.4 (29.0-96.3)	28.6 (3.7-71.0)	14.3 (0.4-57.9)	85.7 (42.1-99.6)	373 (244-713)	225 (125-389)	71.4 (29.0-96.3)	57.1 (18.4-90.1)
2005-2007 (n=11)	36.4 (10.9-69.2)	63.4 (30.8-89.1)	36.4 (10.9-69.2)	9.1 (0.2-41.3)	90.9 (58.7-99.8)	389 (191-594)	207 (191-411)	27.3 (6.0-61.0)	9.1 (0.2-41.3)
2015-2017 (n=26)	19.2 (6.6-39.4)	53.8 (33.4-73.4)	46.2 (26.6-66.6)	19.2 (6.6-39.4)	80.8 (60.6-93.4)	402 (195-710)	286 (195-371)	84.6 (65.1-95.6)	61.5 (40.6-79.8)
3-way p	P=0.04	P=0.36		P=0.59		P=0.88	P=0.39	P=0.08	P=0.24
2-way p	P=0.05	P=0.43		P=0.79		P=0.91	P=0.45	P=0.45	P=0.85
Cardiovascular									
1995-1997 (n=25)	88.0 (68.8-97.5)	100 (86.3-100)	0 (0.0-13.7)	16.0 (4.5-36.1)	84.0 (63.9-95.5)	1370 (652-2322)	985 (367-1555)	44.0 (24.4-65.1)	28.0 (12.1-49.4)
2005-2007 (n=10)	80.0 (44.4-97.5)	100 (69.2-100)	0 (0.0-30.8)	20.0 (2.5-55.6)	80.0 (44.4-97.5)	1724 (469-2339)	1235 (275-1621)	40.0 (12.2-73.8)	10.0 (0.3-44.5)
2015-2017 (n=15)	46.7 (21.3-73.4)	93.3 (68.1-99.8)	6.7 (0.2-31.9)	46.7 (21.3-73.4)	53.3 (26.6-78.7)	5288 (2284-8107)	3232 (1528-4052)	66.7 (38.4-88.2)	66.7 (38.4-88.2)
3-way p	P=0.005	P=0.16		P=0.04		P=0.002	P=0.003	P=0.19	P=0.03
2-way p	P=0.005	P=0.22		P=0.04		P=0.002	P=0.002	P=0.18	P=0.02
Other									
1995-1997 (n=44)	93.2 (81.3-98.6)	93.2 (81.3-98.6)	6.8 (1.4-18.7)	79.5 (64.7-90.2)	20.5 (9.8-35.3)	766 (526-950)	479 (311-617)	31.8 (18.6-47.6)	25.0 (13.2-40.3)
2005-2007 (n=26)	57.7 (36.9-76.6)	84.6 (65.1-95.6)	15.4 (4.4-34.9)	73.1 (52.2-88.4)	26.9 (11.6-47.8)	411 (88-1185)	255 (63-799)	50.0 (29.9-70.1)	34.6 (17.2-55.7)

© 2020 Zhang AD et al. *JAMA Network Open.*

2015-2017 (n=49)	69.4 (54.6-81.7)	91.8 (80.4-97.7)	8.2 (2.3-19.6)	77.6 (63.4-88.2)	22.4 (11.8-36.6)	1108 (136-1930)	721 (88-1114)	30.6 (18.3-45.4)	22.4 (11.8-36.6)
3-way p	P=0.009	P=0.85		P=0.83		P=0.40	P=0.58	P=0.87	P=0.76
2-way p	P=0.004	P=0.81		P=0.82		P=0.33	P=0.51	P=0.90	P=0.78

© 2020 Zhang AD et al. *JAMA Network Open.*

eTable 10. Median Duration of Pivotal Trials Supporting New Drugs and Biologics Approved by the US Food and Drug Administration in 1995-1997, 2005-2007, and 2015-2017, Overall and Stratified by Expected Length of Treatment

Characteristics	Median Duration (IQR), weeks
**Overall**	
1995-1997 (n=401)	11.0 (4.9-24.0)
2005-2007 (n=141)	16.0 (6.0-26.0)
2015-2017 (n=253)	24.0 (12.0-37.6)
3-way p	P<0.001
**Expected Length of Treatment**	
Acute	
1995-1997 (n=158)	4.0 (2.9-6.0)
2005-2007 (n=27)	2.5 (2.0-7.5)
2015-2017 (n=30)	6.0 (2.0-12.0)
3-way p	P=0.55
Intermediate	
1995-1997 (n=39)	24.5 (4.0-52.0)
2005-2007 (n=33)	12.0 (5.2-19.8)
2015-2017 (n=67)	24.0 (12.0-64.1)
3-way p	P=0.06
Chronic	
1995-1997 (n=204)	16.0 (8.0-26.0)
2005-2007 (n=81)	24.0 (12.0-27.3)
2015-2017 (n=156)	24.0 (12.0-26.1)
3-way p	P=0.005

© 2020 Zhang AD et al. *JAMA Network Open.*

eTable 11. Duration of Aggregated Pivotal Trials Supporting New Drug and Biologic Indications Approved by the US Food and Drug Administration in 1995-1997, 2005-2007, and 2015-2017, Overall and Stratified by Expected Length of Treatment

**Characteristics**	**Duration, % (95% CI)**	
	**≥1 Trial of ≥6 Months**	**≥1 Trial of ≥12 Months**
**Overall**		
1995-1997 (n=124)	25.8 (18.4-34.4)	18.5 (12.1-26.5)
2005-2007 (n=63)	34.9 (23.3-48.0)	19.0 (10.2-30.9)
2015-2017 (n=106)	46.2 (36.5-56.2)	35.8 (26.8-45.7)
3-way p	P=0.001	P=0.003
**Expected Length of Treatment**		
Acute		
1995-1997 (n=53)	1.9 (0.0-10.1)	1.9 (0.0-10.1)
2005-2007 (n=15)	6.7 (0.2-31.9)	6.7 (0.2-31.9)
2015-2017 (n=12)	16.7 (2.1-48.4)	16.7 (2.1-48.4)
3-way p	P=0.03	P=0.03
Intermediate		
1995-1997 (n=10)	70.0 (34.8-93.3)	50.0 (18.7-81.3)
2005-2007 (n=15)	26.7 (7.8-55.1)	13.3 (1.7-40.5)
2015-2017 (n=35)	62.9 (44.9-78.5)	45.7 (28.8-63.4)
3-way p	P=0.67	P=0.62
Chronic		
1995-1997 (n=61)	39.3 (27.1-52.7)	27.9 (17.1-40.8)
2005-2007 (n=33)	51.5 (33.5-69.2)	27.3 (13.3-45.5)
2015-2017 (n=59)	42.4 (29.6-55.9)	33.9 (22.1-47.4)
3-way p	P=0.73	P=0.47

© 2020 Zhang AD et al. *JAMA Network Open.*

eTable 12. Characteristics of Pivotal Trials Supporting New Drugs and Biologics Approved by the US Food and Drug Administration in 1995-1997, 2005-2007, and 2015-2017, Stratified by Use of Priority Review and Accelerated Approval

	Random-ized, % (95% CI)	Double-Blinded, % (95% CI)	Comparator, % (95% CI)			Endpoint, % (95% CI)			Median Patients (IQR)		Median Duration (IQR), weeks
			Active	Placebo	None	Clinical	Scale	Surrogate	Overall	Intervention	
**Priority Review**											
Yes											
1995-1997 (n=84)	84.5 (75.0-91.5)	74.1 (64.1-83.2)	36.9 (26.6-48.1)	44.0 (33.2-55.3)	19.0 (11.3-29.1)	20.2 (12.3-30.4)	6.0 (2.0-13.3)	73.8 (63.1-82.8)	236 (94-421)	148 (63-248)	24.0 (16.0-51.3)
2005-2007 (n=62)	75.8 (63.3-85.8)	58.1 (44.8-70.5)	37.1 (25.2-50.3)	38.7 (26.6-51.9)	24.2 (14.2-36.7)	37.1 (25.2-50.3)	8.0 (2.7-17.8)	54.8 (41.7-67.5)	344 (107-616)	230 (107-344)	16.0 (7.8-30.3)
2015-2017 (n=124)	66.1 (57.1-74.4)	53.2 (44.1-62.2)	22.6 (15.6-31.0)	43.5 (34.7-52.7)	33.9 (25.6-42.9)	18.5 (12.1-26.5)	13.7 (8.2-21.0)	67.7 (58.8-75.9)	227 (118-576)	166 (85-357)	24.0 (12.0-50.1)
3-way p	P=0.003	P=0.003	P=0.02	P=1.00	P=0.02	P=0.58	P=0.06	P=0.49	P=0.16	P=0.05	P=0.49
2-way p	P=0.003	P=0.003		P=0.02			P=0.20		P=0.11	P=0.03	P=0.26
No											
1995-1997 (n=317)	96.1 (93.3-98.0)	80.8 (76.0-85.0)	46.1 (40.5-51.7)	48.3 (42.6-53.9)	5.7 (3.4-8.8)	50.0 (44.4-55.6)	8.5 (5.7-12.2)	41.5 (36.0-47.1)	293 (166-454)	185 (97-290)	8.0 (4.0-16.0)
2005-2007 (n=79)	87.3 (78.0-93.8)	74.7 (63.6-83.8)	31.6 (21.6-43.1)	55.7 (44.1-66.9)	12.7 (6.2-22.0)	21.5 (13.1-32.2)	13.9 (7.2-23.5)	64.6 (53.0-75.0)	432 (258-647)	252 (162-402)	12.0 (5.0-26.0)
2015-2017 (n=129)	97.7 (93.3-99.5)	81.4 (73.6-87.7)	35.7 (27.4-44.6)	62.0 (53.1-70.4)	2.3 (0.5-6.6)	27.9 (20.4-36.5)	20.9 (14.3-29.0)	51.1 (42.2-60.1)	549 (441-931)	351 (249-553)	24.0 (12.0-26.1)
3-way p	P=0.94	P=0.94	P=0.02	P=0.007	P=0.40	P<0.001	P<0.001	P=0.02	P<0.001	P<0.001	P<0.001
2-way p	P=0.41	P=0.88		P=0.02			P<0.001		P<0.001	P<0.001	P<0.001
**Accelerated Approval**											
Yes											
1995-1997 (n=34)	82.4 (65.5-93.2)	76.5 (58.8-89.3)	38.2 (22.2-56.4)	44.1 (27.2-62.1)	17.6 (6.8-34.5)	11.8 (3.3-27.5)	0	88.2 (72.5-96.7)	196 (88-357)	124 (60-202)	24.0 (16.0-24.0)
2005-2007 (n=25)	56.0 (34.9-75.6)	28.0 (12.1-49.4)	24.0 (9.4-45.1)	32.0 (14.9-53.5)	44.0 (24.4-65.1)	8.0 (1.0-26.0)	0	92.0 (74.0-99.0)	196 (97-387)	186 (97-256)	20.0 (10.7-24.0)
2015-2017 (n=23)	34.8 (16.4-57.3)	26.1 (10.2-48.4)	0.0 (0.0-14.8)	34.8 (16.4-57.3)	65.2 (42.7-83.6)	0.0 (0.0-14.8)	0	1.00 (85.2-1.00)	129 (87-198)	124 (64-176)	47.4 (31.5-93.7)
3-way p	P<0.001	P<0.001	P<0.001	P=0.43	P<0.001	P=0.10	NA	P=0.10	P=0.26	P=0.86	P=0.003
2-way p	P<0.001	P<0.001		P<0.001			P=0.24		P=0.18	P=0.73	P<0.001
No											
1995-1997 (n=367)	94.7 (91.8-96.8)	79.4 (74.8-83.5)	44.7 (39.5-49.9)	47.7 (42.5-52.9)	7.6 (5.1-10.8)	46.7 (41.5-52.0)	8.7 (6.1-12.1)	44.5 (39.4-49.8)	287 (155-447)	175 (97-284)	8.0 (4.3-24.0)

© 2020 Zhang AD et al. *JAMA Network Open.*

2005-2007 (n=116)	87.9 (80.6-93.2)	75.9 (67.0-83.3)	36.2 (27.5-45.6)	51.7 (42.3-61.1)	12.1 (6.8-19.4)	32.8 (24.3-42.1)	13.4 (8.1-21.4)	53.4 (44.0-62.8)	463 (243-652)	259 (151-399)	12.0 (5.0-26.0)
2015-2017 (n=230)	87.0 (81.9-91.0)	71.7 (65.4-77.5)	32.2 (26.2-38.6)	54.8 (48.1-61.3)	13.0 (9.0-18.1)	25.7 (20.1-31.8)	19.1 (14.3-24.8)	55.2 (48.5-61.8)	496 (267-741)	307 (164-473)	24.0 (12.0-26.0)
3-way p	P<0.001	P=0.03	P=0.002	P=0.09	P=0.03	P<0.001	P<0.001	P=0.009	P<0.001	P<0.001	P<0.001
2-way p	P<0.001	P=0.03	P=0.004			P<0.001			P<0.001	P<0.001	P<0.001

© 2020 Zhang AD et al. *JAMA Network Open.*

eTable 13. Characteristics of Aggregated Pivotal Trials Supporting New Drugs and Biologic Indications Approved by the US Food and Drug Administration in 1995-1997, 2005-2007, and 2015-2017, Stratified by Use of Priority Review and Accelerated Approval

	≥2 Trials (%, 95% CI)	Comparators (%, 95% CI)		Endpoints (%, 95% CI)		Median Patients in Aggregated Pivotal Efficacy Trials (IQR)		Duration (%, 95% CI)	
		≥1 Trial Using Any Comparator	Single-Arm Only	≥1 Trial Using Clinical-Type Endpoints	Surrogate Endpoints Only	Overall	Intervention	≥1 Trial of ≥6 Months	≥1 Trial of ≥12 Months
**Priority Review**									
Yes									
1995-1997 (n=31)	77.4 (58.9-90.4)	87.1 (40.2-96.4)	12.9 (3.6-29.8)	35.5 (19.2-54.6)	64.5 (45.4-80.8)	586 (215-949)	380 (142-569)	48.4 (30.2-66.9)	29.0 (14.2-48.0)
2005-2007 (n=34)	55.9 (37.9-72.8)	79.4 (62.1-91.3)	20.6 (8.7-37.9)	47.1 (29.8-64.9)	52.9 (35.1-70.2)	601 (163-1055)	396 (161-568)	38.2 (22.2-56.4)	26.5 (12.9-44.4)
2015-2017 (n=60)	38.3 (26.1-51.8)	71.7 (58.6-82.5)	28.3 (17.5-41.4)	43.3 (30.6-56.8)	56.7 (43.2-69.4)	360 (135-949)	253 (100-669)	48.3 (35.2-61.6)	36.7 (24.6-50.1)
3-way p	P<0.001	P=0.09		P=0.55		P=0.27	P=0.38	P=0.87	P=0.39
2-way p	P<0.001	P=0.10		P=0.48		P=0.24	P=0.38	P=1.00	P=0.47
No									
1995-1997 (n=93)	81.7 (72.3-89.0)	98.9 (94.2-100)	1.1 (0.0-5.8)	68.8 (58.4-78.0)	31.1 (22.0-41.6)	811 (532-1397)	513 (297-961)	18.3 (11.0-27.6)	15.1 (8.5-24.0)
2005-2007 (n=29)	65.5 (45.7-82.1)	96.6 (82.2-99.9)	3.4 (0.0-17.8)	51.7 (32.5-70.6)	48.3 (29.4-67.5)	1005 (328-1733)	661 (223-1165)	31.0 (15.3-50.8)	10.3 (2.2-27.4)
2015-2017 (n=46)	71.7 (56.5-84.0)	97.8 (88.5-99.9)	2.2 (0.0-11.5)	69.6 (54.2-82.3)	30.4 (17.7-45.8)	1646 (1043-2820)	1019 (543-1589)	43.5 (28.9-58.9)	34.8 (21.4-50.2)
3-way p	P=0.13	P=0.57		P=0.86		P<0.001	P=0.001	P=0.002	P=0.01
2-way p	P=0.18	P=0.62		P=0.93		P<0.001	P<0.001	P=0.002	P=0.008
**Accelerated Approval**									
Yes									
1995-1997 (n=12)	75.0 (42.8-94.5)	91.7 (61.5-99.8)	8.3 (0.2-38.5)	16.7 (2.1-48.4)	83.3 (51.6-97.9)	756 (399-988)	433 (230-558)	33.3 (9.9-65.1)	16.7 (2.1-48.4)
2005-2007 (n=13)	69.2 (38.6-90.9)	69.2 (38.6-90.9)	30.8 (9.1-61.4)	7.7 (0.2-36.0)	92.3 (64.0-99.8)	461 (195-658)	261 (195-483)	30.8 (9.1-61.4)	23.1 (5.0-53.8)
2015-2017 (n=13)	30.8 (9.1-61.4)	30.8 (9.1-61.4)	69.2 (38.6-90.9)	0 (0.0-24.7)	100 (75.3-100)	196 (138-228)	145 (109-221)	76.9 (46.2-95.0)	46.2 (19.2-74.9)
3-way p	P=0.02	P=0.002		P=0.12		P<0.001	P<0.001	P=0.03	P=0.10
2-way p	P=0.03	P=0.003		P=0.15		P<0.001	P=0.002	P=0.03	P=0.13
No									
1995-1997 (n=112)	81.3 (72.8-88.0)	96.4 (91.1-99.0)	3.6 (1.0-8.9)	65.2 (55.6-73.9)	34.8 (26.1-44.4)	774 (479-1370)	501 (247-851)	25.0 (17.3-34.1)	18.8 (12.0-27.2)

© 2020 Zhang AD et al. *JAMA Network Open.*

2005-2007 (n=50)	58.0 (43.2-71.8)	92.0 (80.8-97.8)	8.0 (2.2-19.2)	60.0 (45.2-73.6)	40.0 (26.4-54.8)	831 (252-1564)	435 (145-915)	36.0 (22.9-50.8)	18.0 (8.6-31.4)
2015-2017 (n=93)	55.9 (45.2-66.2)	90.3 (82.4-95.5)	9.7 (4.5-17.6)	62.4 (51.7-72.2)	37.6 (27.8-48.3)	1079 (342-2390)	612 (241-1365)	41.9 (31.8-52.6)	34.4 (24.9-45.0)
3-way p	P<0.001	P=0.08		P=0.66		P=0.15	P=0.19	P=0.01	P=0.01
2-way p	P<0.001	P=0.08		P=0.68		P=0.14	P=0.16	P=0.01	P=0.01

© 2020 Zhang AD et al. *JAMA Network Open.*

eTable 14. Characteristics of Pivotal Trials Supporting New Drugs and Biologics Approved by the US Food and Drug Administration in 1995-1997, 2005-2007, and 2015-2017, Stratified by Use of Any Special Regulatory Program, Considering Orphan Designation as a Special Regulatory Program

	**Random-ized, % (95% CI)**	**Double-Blinded, % (95% CI)**	**Comparator, % (95% CI)**			**Endpoint, % (95% CI)**			**Median Patients in Pivotal Efficacy Trials (IQR)**		**Median Duration (IQR), weeks**
			**Active**	**Placebo**	**None**	**Clinical**	**Scale**	**Surrogate**	**Overall**	**Intervention**	
**Special Regulatory Program**											
Any											
1995-1997 (n=96)	83.3 (74.4-90.2)	72.0 (61.8-80.9)	36.5 (26.9-46.9)	43.8 (33.6-54.3)	19.8 (12.4-29.2)	24.0 (15.8-33.7)	7.3 (3.0-14.4)	68.8 (58.5-77.8)	236 (94-425)	145 (63-259)	24.0 (15.0-52.3)
2005-2007 (n=64)	75.0 (62.6-85.0)	56.3 (43.3-68.6)	35.9 (24.3-48.9)	39.1 (27.1-52.1)	25.0 (15.0-37.4)	37.5 (25.7-50.5)	7.8 (2.6-17.3)	54.7 (41.7-67.2)	329 (115-605)	225 (106-336)	17.0 (9.7-28.3)
2015-2017 (n=133)	68.4 (59.8-76.2)	55.6 (46.8-64.2)	22.6 (15.8-30.6)	45.9 (37.2-54.7)	31.6 (23.8-40.2)	19.5 (13.2-26.3)	14.3 (8.8-21.4)	66.2 (57.5-74.1)	227 (119-655)	167 (84-366)	24.0 (12.0-49.3)
3-way p	P=0.010	P=0.016	P=0.019	P=0.70	P=0.04	P=0.34	P=0.08	P=0.79	P=0.10	P=0.03	P=0.58
2-way p	P=0.011	P=0.012	P=0.03			P=0.23			P=0.08	P=0.02	P=0.33
None											
1995-1997 (n=305)	97.0 (94.3-98.6)	81.7 (76.8-85.9)	46.6 (40.9-52.3)	48.5 (42.8-54.3)	4.9 (2.8-8.0)	50.0 (44.2-55.8)	8.2 (5.4-11.9)	41.8 (36.2-47.5)	297 (173-450)	186 (100-289)	8.0 (4.0-15.7)
2005-2007 (n=77)	88.3 (79.0-94.5)	76.6 (65.6-85.5)	32.5 (22.2-44.1)	55.8 (44.1-67.2)	11.7 (5.5-21.0)	20.8 (12.4-31.5)	14.3 (7.4-24.1)	64.9 (53.2-75.5)	455 (273-651)	254 (163-409)	12.0 (5.0-26.0)
2015-2017 (n=120)	97.5 (92.9-99.5)	80.8 (72.6-87.4)	36.7 (28.1-45.9)	60.8 (51.5-69.6)	2.5 (0.5-7.1)	27.5 (19.7-36.4)	20.8 (14.0-29.2)	51.7 (42.4-60.9)	548 (446-958)	344 (254-580)	24.0 (12.0-26.0)
3-way p	P=0.69	P=0.71	P=0.03	P=0.018	P=0.63	P<0.001	P<0.001	P=0.016	P<0.001	P<0.001	P<0.001
2-way p	P=0.77	P=0.84	P=0.06			P<0.001			P<0.001	P<0.001	P<0.001

© 2020 Zhang AD et al. *JAMA Network Open.*

eTable 15. Characteristics of Aggregated Pivotal Trials Supporting New Drugs and Biologic Indications Approved by the US Food and Drug Administration in 1995-1997, 2005-2007, and 2015-2017, Stratified by Use of Any Special Regulatory Program, Considering Orphan Designation as a Special Regulatory Program

	**≥2 Trials (%, 95% CI)**	**Comparators (%, 95% CI)**		**Endpoints (%, 95% CI)**		**Median Patients in Aggregated Pivotal Efficacy Trials (IQR)**		**Duration (%, 95% CI)**	
		**≥1 Trial Using Any Comparator**	**Single-Arm Only**	**≥1 Trial Using Clinical Endpoints or Clinical Scales**	**Trials Using Surrogate Endpoints Only**	**Overall**	**Intervention**	**≥1 Trial of ≥6 Months**	**≥1 Trial of ≥12 Months**
**Special Regulatory Program**									
Any									
1995-1997 (n=35)	77.1 (59.9-89.6)	88.6 (73.3-96.8)	11.4 (3.2-26.7)	40.0 (23.9-57.9)	60.0 (42.1-76.1)	586 (244-949)	380 (151-569)	48.6 (31.4-66.0)	31.4 (16.9-49.3)
2005-2007 (n=36)	52.8 (35.5-69.6)	77.8 (60.8-89.9)	22.2 (10.1-39.2)	47.2 (30.4-64.5)	52.8 (35.5-69.6)	534 (166-979)	340 (119-544)	36.1 (20.8-53.8)	25.0 (12.1-42.2)
2015-2017 (n=68)	35.3 (24.1-47.8)	75.0 (63.0-84.7)	25.0 (15.3-37.0)	45.6 (33.5-58.1)	54.4 (41.9-66.5)	360 (129-1071)	253 (89-574)	48.6 (36.2-61.0)	35.3 (24.1-47.8)
3-way p	P<0.001	P=0.12		P=0.64		P=0.22	P=0.32	P=0.83	P=0.57
2-way p	P<0.001	P=0.11		P=0.59		P=0.16	P=0.27	P=1.00	P=0.70
None									
1995-1997 (n=89)	82.0 (72.5-89.4)	98.9 (93.9-100)	1.1 (0.0-6.1)	68.5 (57.8-78.0)	31.5 (22.0-42.2)	811 (535-1397)	514 (308-961)	16.9 (9.8-26.3)	13.5 (7.2-22.4)
2005-2007 (n=27)	70.4 (49.8-86.2)	100 (87.2-100)	0.0 (0.0-12.8)	51.9 (31.9-71.3)	48.1 (28.7-68.1)	1167 (419-1835)	680 (244-1374)	33.3 (16.5-54.0)	11.1 (2.4-29.2)
2015-2017 (n=38)	84.2 (68.7-94.0)	97.4 (86.2-99.9)	2.6 (0.0-13.8)	71.1 (54.1-84.6)	28.9 (15.4-45.9)	1833 (1207-3180)	1089 (715-1887)	42.1 (26.3-59.2)	36.8 (21.8-54.0)
3-way p	P=0.99	P=0.58		P=0.96		P<0.001	P<0.001	P=0.002	P=0.005
2-way p	P=0.77	P=0.54		P=0.78		P<0.001	P<0.001	P=0.003	P=0.003

© 2020 Zhang AD et al. *JAMA Network Open.*
